# The Transcription Factor TFII-I Promotes DNA Translesion Synthesis and Genomic Stability

**DOI:** 10.1371/journal.pgen.1004419

**Published:** 2014-06-12

**Authors:** Farjana J. Fattah, Kodai Hara, Kazi R. Fattah, Chenyi Yang, Nan Wu, Ross Warrington, David J. Chen, Pengbo Zhou, David A. Boothman, Hongtao Yu

**Affiliations:** 1 Department of Pharmacology, University of Texas Southwestern Medical Center, Dallas, Texas, United States of America; 2 Simmons Comprehensive Cancer Center, University of Texas Southwestern Medical Center, Dallas, Texas, United States of America; 3 Howard Hughes Medical Institute, Chevy Chase, Maryland, United States of America; 4 Division of Molecular Radiation Biology, Department of Radiation Oncology, University of Texas Southwestern Medical Center, Dallas, Texas, United States of America; 5 Department of Pathology and Laboratory Medicine, Weill Medical College of Cornell University, New York, New York, United States of America; SUNY Stony Brook, United States of America

## Abstract

Translesion synthesis (TLS) enables DNA replication through damaged bases, increases cellular DNA damage tolerance, and maintains genomic stability. The sliding clamp PCNA and the adaptor polymerase Rev1 coordinate polymerase switching during TLS. The polymerases Pol η, ι, and κ insert nucleotides opposite damaged bases. Pol ζ, consisting of the catalytic subunit Rev3 and the regulatory subunit Rev7, then extends DNA synthesis past the lesion. Here, we show that Rev7 binds to the transcription factor TFII-I in human cells. TFII-I is required for TLS and DNA damage tolerance. The TLS function of TFII-I appears to be independent of its role in transcription, but requires homodimerization and binding to PCNA. We propose that TFII-I bridges PCNA and Pol ζ to promote TLS. Our findings extend the general principle of component sharing among divergent nuclear processes and implicate TLS deficiency as a possible contributing factor in Williams-Beuren syndrome.

## Author Summary

DNA translesion synthesis (TLS) allows the DNA replication machinery to replicate past damaged bases, thus increasing cellular tolerance for DNA damage and maintaining genomic stability. Suppression of TLS is expected to enhance the efficacy of the anti-cancer drug, cisplatin. TLS employs a special set of DNA polymerases, including Pol ζ. The TLS polymerases are also involved in somatic hypermutation and proper immune response in mammals. Thus, it is critical to understand the underlying mechanisms of TLS. In this study, we have discovered the transcription factor TFII-I as a new Pol ζ-binding protein in human cells. We show that TFII-I is indeed required for TLS and DNA damage tolerance. We further delineate the mechanism by which TFII-I contributes to TLS. Our study significantly advances the molecular understanding of TLS, and provides a fascinating example of component sharing among disparate nuclear processes. Finally, because one copy of the TFII-I gene is deleted in Williams-Beuren syndrome (WBS), our work implicates TLS deficiency as a potential causal factor of this human genetic disorder.

## Introduction

DNA bases experience many types of damage caused by both endogenous and exogenous factors. DNA repair pathways, such as the global genomic nucleotide excision repair (GG-NER) pathway, actively remove damaged bases [1]. In addition, when damaged bases are not completely removed, DNA translesion synthesis (TLS) allows replication past these lesions, thus increasing DNA damage tolerance and maintaining genomic integrity [2]. TLS requires a set of specialized DNA polymerases, including the Y family polymerases, Rev1, Pol η, ι, and κ, and the B family polymerase Pol ζ containing the Rev3 catalytic subunit and the Rev7 regulatory subunit [2]. Certain TLS polymerases, including Pol ζ, are involved in somatic hypermutation of immunoglobulin genes [3]–[5].

Recent advances have established that multiple polymerase-switching events occur during TLS, and have begun to elucidate the elaborate molecular mechanisms that regulate these steps. When replicative polymerases encounter damaged DNA bases, such as those crosslinked by UV, the sliding clamp PCNA is ubiquitinated [6]. Ubiquitinated PCNA recruits Pol η, ι, or κ through the adaptor polymerase Rev1 [7]–[13]. Pol η, ι, or κ inserts nucleotides directly opposite to the DNA lesion [14]–[16]. In a poorly understood second switch, Pol ζ is employed to extend DNA replication past the lesion. Rev1 can simultaneously bind to Pol ζ and one of the Y family polymerases, Pol η, ι, or κ, suggesting that this polymerase switching step might occur through simple repositioning of a large, multi-polymerase assembly on the DNA template [17]. After TLS is completed, replicative DNA polymerases re-engage with PCNA and resume high-fidelity replication.

TFII-I was first identified as a general transcription factor that bound to a pyrimidine-rich Initiator (Inr) sequence at the transcription start site and supported transcription in an *in vitro* reconstituted system [18]. TFII-I contains an N-terminal dimerization domain, six repeated domains (called R1-R6), and four AlkB homologue 2 PCNA-interacting motifs (APIM) motifs, among other features [19], [20]. Recent studies have suggested that TFII-I is not a general transcription factor required for all Inr-dependent transcription [21]. Instead, it has signal- and context-dependent regulatory roles in the transcription of specific genes. Interestingly, TFII-I is one of 26–28 genes affected by a hemizygous deletion of the chromosome 7q11.23 region in the rare human disorder, Williams-Beuren syndrome (WBS) [22]. WBS patients exhibit a wide spectrum of phenotypes, including distinctive craniofacial features, cardiovascular abnormalities, and mental retardation. Heterozygous mutant mice with the N-terminal 140 residues of TFII-I deleted show WBS-like craniofacial and neurobehavioral alterations, linking this region of TFII-I to a subset of WBS phenotypes [23].

In this study, we show that TFII-I physically interacts with the Pol ζ subunit Rev7 (also known as Mad2B). Functional studies reveal that TFII-I is indeed required for TLS and DNA damage tolerance in human cells. Depletion of TFII-I affects the transcription of a small number of genes, none of which are known to be involved in TLS, suggesting that the TLS function of TFII-I is independent of its role in transcription. Instead, both PCNA binding and homodimerization of TFII-I are required for TLS. We propose that TFII-I connects Pol ζ to PCNA and facilities TLS. Because a TFII-I mutant lacking its N-terminal dimerization domain is defective in TLS, our findings also implicate TLS deficiency as a potential contributing factor of WBS.

## Results

### Identification of TFII-I as a Rev7-binding protein in human cells

**Figure 1:**
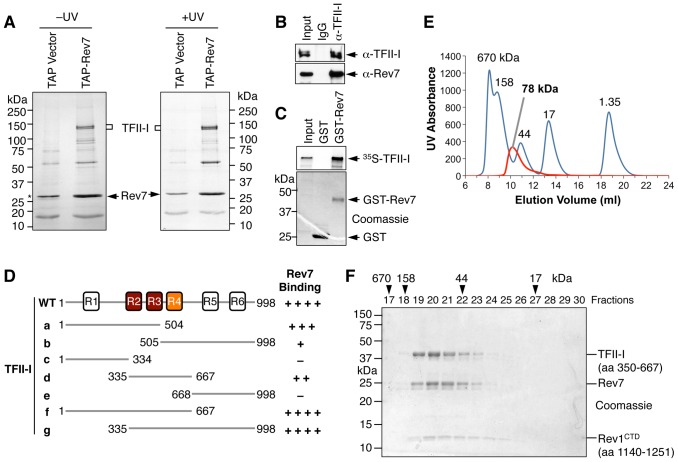
TFII-I is a novel Pol ζ-binding protein. (A) TAP-Rev7 complexes from 293T cells without (−) or with UV-C (254 nm) irradiation (10 J/m^2^) were purified in separate experiments, analyzed with SDS-PAGE, and stained with Coomassie. The doublet bands belonging to TFII-I are labeled. Note that cleaved TAP-Rev7 co-migrated with a contaminating band at 28 kDa. (B) Lysates of parental 293T cells (without TAP-Rev7 expression) and the IgG or anti-TFII-I immunoprecipitates (IP) were blotted with anti-TFII-I or anti-Rev7 antibodies. (C) ^35^S-TFII-I in rabbit reticulocyte lysate (Input) or bound to GST or GST-Rev7 beads were separated by SDS-PAGE and analyzed with a phosphor imager (top panel) or stained with Coomassie (bottom panel). (D) Schematic drawing of TFII-I and its fragments. The six TFII-I repeats R1-R6 are indicated. The Rev7-binding activities of various TFII-I fragments are shown on the right. (E) Purified recombinant TFII-I (residues 350–667), the Rev7 R124A–Rev3L (residues 1847–1898) complex, and Rev1 CTD were mixed at 1∶1∶1 molar ratios and fractioned on a Superdex 200 column. The UV traces of the complex (red) and the molecular mass standards (in blue) are shown. The calculated native molecular mass of the complex is indicated. (F) The indicated column fractions in (E) were separated by SDS-PAGE and stained with Coomassie. The eluting positions of the native molecular mass standards are indicated by arrowheads.

Rev7 shares sequence similarity with the spindle checkpoint protein Mad2 [24]. Both contain a HORMA (Hop1-Rev7-Mad2) domain that mediates protein–protein interactions [25]. Because of our long-standing interest in the structure and function of Mad2 [26]–[28], we examined the function and regulation of Rev7. We created 293T cell lines stably expressing human Rev7 fused at its N-terminus with a tandem affinity purification (TAP) tag and purified TAP-Rev7 complexes from these cells with or without UV irradiation (10 J/m^2^). TAP-Rev7 preparations from both samples contained prominent doublet bands at 140 kDa (Figure 1A). Mass spectrometry analysis of the unirradiated sample revealed that these bands belonged to the transcription factor, TFII-I, which was known to have multiple alternative splicing variants (Table S1). The sequence coverage of TFII-I was 52.4%. In addition to TFII-I, we also identified the known Rev7-binding protein, ZNF828/CAMP [29], with a sequence coverage of 13.6%. Another potential Rev7-binding protein was CAD (Carbamoyl-phosphate synthetase 2, Aspartate transcarbamylase, and Dihydroorotase), a key multifunctional enzyme in the pyrimidine biosynthetic pathway, suggesting a possible link between TLS and pyrimidine biosynthesis. Rev3L was not detected in Rev7 preparations, presumably due to its low abundance in cells.

Because TFII-I was the most abundant Rev7-binding protein in the TAP-Rev7 samples, we focused on the Rev7–TFII-I interaction in this study. Endogenous Rev7 and TFII-I proteins interacted with each other in human cells (Figure 1B). TFII-I did not interact with Pol ι or Pol η (Figure S1A). Recombinant GST-Rev7 bound to *in vitro* translated TFII-I (Figure 1C). A minimal Rev7-binding domain of TFII-I was mapped to its middle region containing R2-R4 repeats (Figure 1D and Figure S1). This minimal TFII-I domain, however, bound more weakly to Rev7 than the full-length TFII-I did, suggesting that additional regions of TFII-I might contribute to Rev7 binding. These results suggested that Rev7 physically interacted with TFII-I.

As a regulatory subunit of Pol ζ, Rev7 simultaneously binds to a small Rev7-binding motif (RBM) in Rev3L and the C-terminal domain (CTD) of Rev1, thus bridging an interaction between Rev1 and Rev3L (Figure S2A) [17]. When bound to Rev3L, Rev7 adopts the closed conformation and traps Rev3L RBM with a topological embrace through its “seat belt” [30]. The Rev1 CTD binds Rev7 at a site opposite of the Rev3L-binding site [17], [31]. We next tested whether TFII-I binding to Rev7 was compatible with Rev3L–Rev7 or Rev1–Rev7 interactions. A recombinant purified TFII-I fragment (residues 350–667) containing R2-R4 co-fractionated with Rev7 bound to Rev3L RBM (residues 1847–1898) with Rev1 CTD (residues 1140–1251) (Figure 1E and 1F). Based on gel filtration, the native molecular mass of this miniature TFII-I–Rev7–Rev3L–Rev1 complex was 78 kDa, which was consistent with the formation of a 1∶1∶1∶1 heterotetramer with an expected molecular mass of 81 kDa. With the small amount of each protein loaded, the Rev3L fragment was not visible by Coomassie blue staining. This fragment could only be visualized with large amounts of proteins loaded (Figure S2B). Thus, our results suggest that TFII-I can bind to the Rev3L–Rev7–Rev1 complex *in vitro*.

### TFII-I is required for DNA damage tolerance

**Figure 2:**
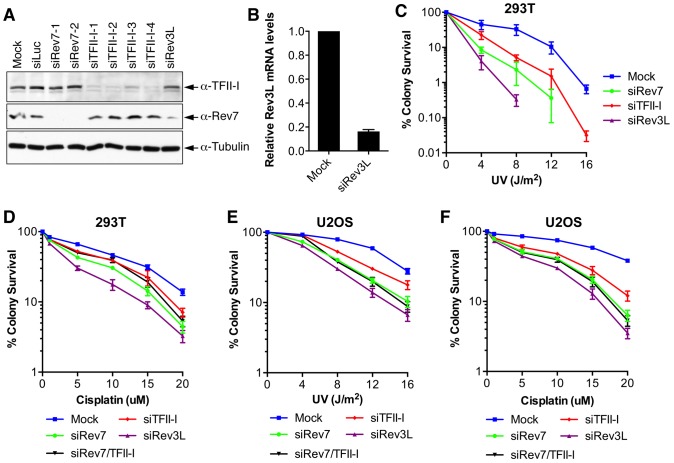
TFII-I depletion in human cells causes UV and cisplatin sensitivity. (A) Lysates of 293T cells transfected with the indicated siRNAs were blotted with indicated antibodies. (B) Quantitative PCR analysis of the Rev3L mRNA levels in mock or siRev3L transfected 293T cells. (C) Colony survival curves of 293T cells that were transfected with the indicated siRNAs and irradiated with increasing doses of UV. The mean and standard deviation (SD) of three independent experiments are shown. (D) Colony survival curves of 293T cells that were transfected with the indicated siRNAs and increasing doses of cisplatin. The mean and standard deviation (SD) of three independent experiments are shown. (E) Colony survival curves of U2OS cells that were transfected with the indicated siRNAs and irradiated with increasing doses of UV. The mean and standard deviation (SD) of three independent experiments are shown. (F) Colony survival curves of U2OS cells that were transfected with the indicated siRNAs and increasing doses of cisplatin. The mean and standard deviation (SD) of three independent experiments are shown.

Inactivation of either subunit of Pol ζ, Rev3L or Rev7, reduces colony formation of mammalian cells treated with UV and cisplatin, presumably because they are required to bypass DNA damage induced by these agents [32]–[34]. We first confirmed that human 293T and U2OS cells depleted of Rev3L or Rev7 with small interfering RNA (siRNA) were indeed sensitive to UV or cisplatin using colony formation assays (Figure 2). Because antibodies that could detect endogenous Rev3L were unavailable, the efficiency of Rev3L depletion was indirectly inferred from the reduction of Rev3L mRNA as measured by quantitative PCR (Figure 2B). Depletion of TFII-I similarly resulted in UV and cisplatin sensitivity (Figure 2). Importantly, depletion of both TFII-I and Rev7 did not cause more severe phenotypes than depletion of either one alone did, suggesting that TFII-I might be required for DNA damage tolerance. Efficient depletion of Rev7 and TFII-I was confirmed by Western blots. There were no discernable cell cycle defects in cells depleted of TFII-I, Rev3L, or Rev7 in the absence of UV (Figure S3 and data not shown).

**Figure 3:**
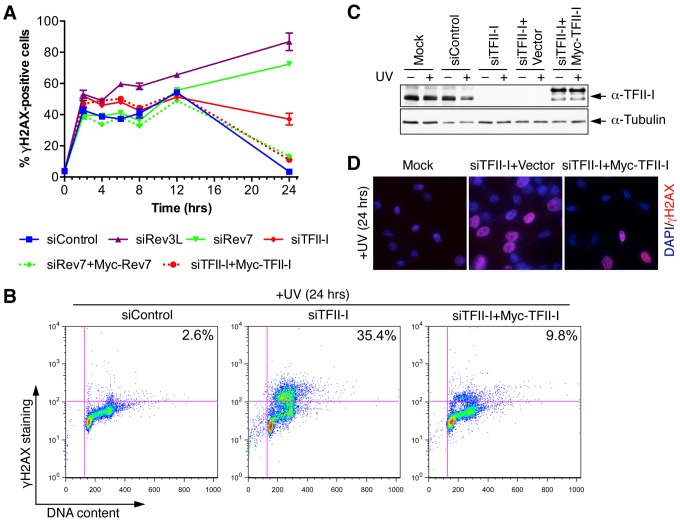
TFII-I is required for DNA damage tolerance in human cells. (A) HeLa Tet-On cells were transfected with the indicated siRNAs and plasmids, treated with UV (10 J/m^2^), and harvested at various timepoints for flow cytometry. The percentage of γ-H2AX-positive cells was plotted against the time after UV irradiation. The means and SDs of two experiments are shown. (B) Representative dot plots of the flow cytometry analysis of cells in (A). (C) Lysates of cells in (A) were blotted with anti-TFII-I and anti-tubulin antibodies. (D) HeLa Tet-On cells were either mock transfected or transfected with siTFII-I along with a control vector or the Myc-TFII-I plasmid and irradiated with UV (10 J/m^2^). At 24 h after UV treatment, cells were fixed and stained with DAPI (blue) and anti-γ-H2AX antibody (red).

To obtain additional evidence for a role of TFII-I in DNA damage tolerance, we stained control and TFII-I RNAi cells for γ-H2AX, a DNA double-strand break (DSB) marker, at different times following the treatment of low-dose UV, and performed flow cytometry analysis. UV irradiation induced DNA damage in all samples. UV-induced DNA damage is expected to stall replication forks in S phase and indirectly produce DSBs. About 40% of all groups of cells were in S phase and positive for γ-H2AX staining at 2 hrs following UV treatment (Figure 3A and S3). At 12 hrs, the majority of these cells were γ-H2AX-positive and blocked in S phase (Figure S3). At 24 hrs after UV irradiation, few siControl cells were γ-H2AX-positive, indicating that they had progressed through S phase and effectively repaired their damaged DNA (Figure 3A and S3). In contrast, the majority of cells depleted of Rev3L or Rev7 remained blocked in S phase, and were γ-H2AX-positive (Figure S3), consistent with a known role of Pol ζ in DNA damage tolerance. Cells depleted of TFII-I were also less efficient in passing through S phase and in repairing DNA damage, as about 40% of TFII-I RNAi cells had positive γ-H2AX staining at 24 hrs after UV irradiation (Figure 3A, B). Most cells positive for γ-H2AX had DNA contents between 2C and 4C, indicating that they were blocked in S phase. Importantly, ectopic expression of siRNA-resistant Myc-TFII-I transgene at levels comparable to that of the endogenous TFII-I largely rescued the S phase block and DNA damage of TFII-I RNAi cells (Figure 3A-D), based on both flow cytometry and γ-H2AX immunostaining. These results indicate that, like Pol ζ, TFII-I is required for DNA damage tolerance, S phase progression, and genomic stability. Pol ζ has recently been suggested to play a direct role in DSB repair through homologous recombination [35]. Our results cannot distinguish between a direct role for TFII-I and Pol ζ in DSB repair and an indirect role for them in DNA repair through supporting TLS and DNA damage tolerance. In the future, it will be interesting to test whether TFII-I is directly involved in DSB repair.

### TFII-I is required for translesion synthesis (TLS)

**Figure 4:**
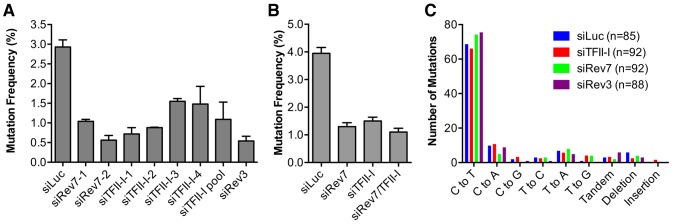
TFII-I is required for DNA translesion synthesis (TLS) in human cells. (A) The mutation frequency of UV-treated *SupF* plasmid in 293T cells transfected with the indicated siRNAs. The mean and SD of three experiments are shown. (B) The mutation frequency of UV-treated *SupF* plasmid in 293T cells transfected with the indicated siRNAs. The mean and SD of two experiments are shown. (C) The mutation spectra of the UV-irradiated *SupF* gene recovered from 293T cells transfected with the indicated siRNAs. The number of clones sequenced for each group is shown in parentheses.

We directly tested whether TFII-I was required for translesion synthesis. To do so, we performed a mutation frequency assay on the UV-irradiated *SupF* shuttle vector plasmid pSP189 [36]. As expected, depletion of Rev3L or Rev7 greatly reduced the mutation frequency of the *SupF* region in the UV-damaged shuttle vector (Figure 4A), consistent with their known roles in TLS. Depletion of TFII-I with multiple siRNAs also reduced the mutation frequency of *SupF*. Importantly, depletion of both TFII-I and Rev7 did not produce a stronger phenotype as did the depletion of either protein alone (Figure 4B), suggesting that TFII-I might work in the same pathway as Pol ζ.

DNA sequencing of the mutated *SupF* clones revealed that inactivation of TFII-I or Pol ζ did not alter the mutation spectrum (Figure 4C and Figure S4). In all samples, the majority of mutations were C∶G to T∶A transitions. Thus, TFII-I depletion reduces TLS efficiency in human cells. We note that there might be subtle differences in the mutation hotspots among different samples (Figure S4). In addition, mutations involving large deletions appeared to be absent in the siTFII-I cells. The significance and the underlying reasons for these apparent differences are unclear at present.

### Depletion of TFII-I or Rev7 does not cause gross transcriptional defects

**Figure 5:**
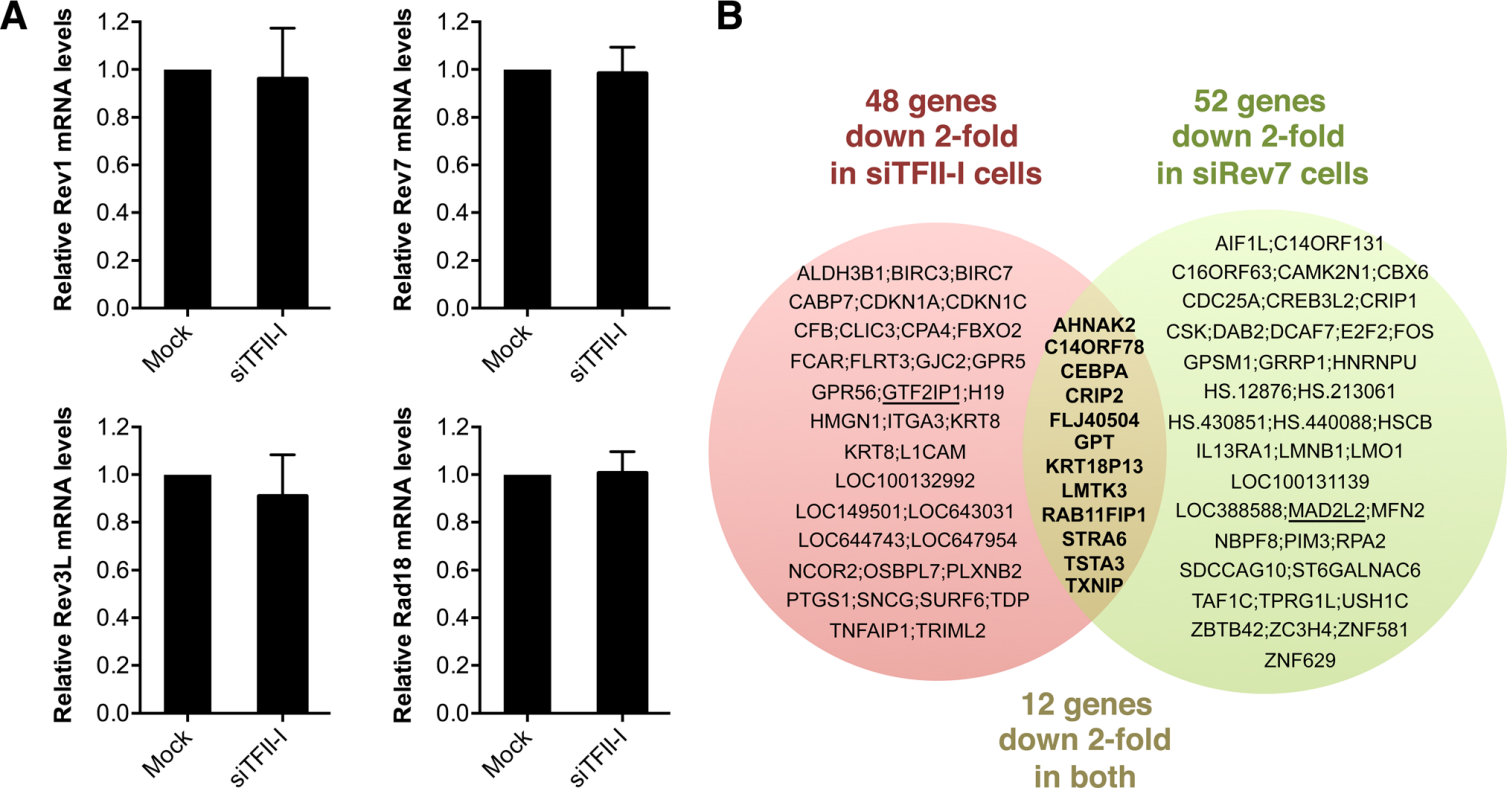
Depletion of TFII-I or Rev7 does not grossly impact transcription. (A) Quantitative PCR analysis of the mRNA levels of Rev1, Rev7, Rev3L, and Rad18 in mock or siTFII-I transfected 293T cells. The mean and standard deviation of triplicate samples are shown. (B) Venn diagram of genes whose expression was down 2-fold or more in HeLa Tet-On cells depleted of TFII-I or Rev7, as determined by microarrays. The bold text highlights the genes whose expression is affected in both sets of cells. GTF2IP1 and Mad2L2 encode TFII-I and Rev7, respectively, and are underlined. Their mRNAs are expected to be depleted by the siRNAs, and serve as positive controls.

Because TFII-I has known functions in transcription, we tested whether the TLS defects of TFII-I RNAi cells were indirectly caused by a defect in the transcription of TLS genes. Using quantitative PCR, we first showed that TFII-I depletion did not substantially alter the mRNA levels of Rev1, Rev7, Rev3L, and Rad18, genes known to be involved in TLS (Figure 5A). Next, we performed gene expression profiling of HeLa Tet-On cells transfected with siControl, siTFII-I, or siRev7. Depletion of TFII-I reduced by two-fold the mRNA levels of only 48 genes (Figure 5B). None of these genes are known to be involved in TLS. Therefore, the TLS deficiency caused by TFII-I RNAi is not an indirect consequence of gross transcriptional defects, although we cannot rule out the possibility that subtle transcriptional defects of multiple genes cumulatively impact TLS. The fact that TFII-I depletion only affects the transcription of so few genes in HeLa cells is not surprising, as many TFII-I target genes are involved in neuronal functions or immune response [37]. Furthermore, two other TFII-I related genes, GTF2IRD1 and GTF2IRD2, might have compensated for the partial loss of TFII-I.

Likewise, depletion of Rev7 only decreased the mRNA levels of about 50 genes (Figure 5B). Moreover, only 12 genes were commonly suppressed in both siTFII-I and siRev7 cells. Therefore, Rev7 does not appear to have a major role in transcription. The primary function of the Rev7–TFII-I interaction is unlikely to be transcriptional regulation in HeLa cells.

### TFII-I bridges the interaction between PCNA and Rev7 in UV-irradiated cells

**Figure 6:**
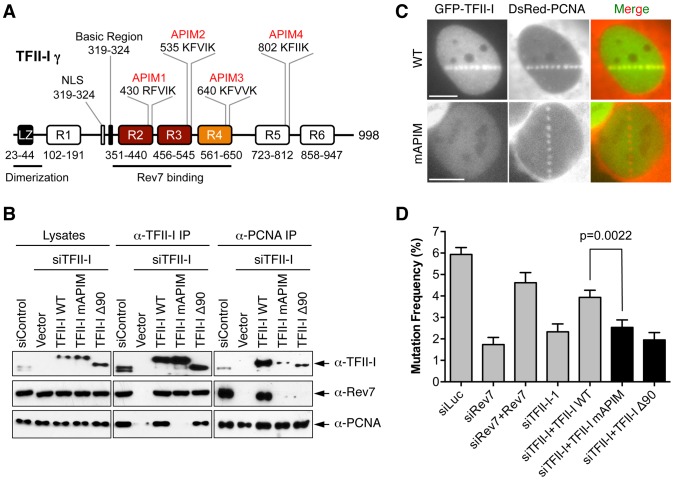
The PCNA-binding motifs and N-terminal region of TFII-I are required for translesion synthesis. (A) Schematic drawing of the domains and motifs of human TFII-I γ. LZ, leucine zipper. (B) U2OS cells were transfected with siControl or siTFII-I along with the indicated TFII-I plasmids, irradiated with UV (60 J/m^2^), and treated with formaldehyde. Lysates, anti-TFII-I IP, and anti-PCNA IP of these cells were blotted with the indicated antibodies. (C) U2OS cells were transfected with GFP-TFII-I wild type (WT) or mAPIM and DsRed-PCNA plasmids, and micro-irradiated with a 365-nm laser along straight lines. GFP and DsRed channels are shown separately in gray scale and together in merge images. Scale bars, 10 µm. (D) The mutation frequency of the UV-treated *SupF* plasmid in 293T cells transfected with the indicated siRNAs and plasmids. The mean and SD of six experiments are shown.

We next explored the mechanism by which TFII-I contributed to TLS. TFII-I contains four APIM motifs [20], [38], which mediates its binding to PCNA (Figure 6A). Because PCNA has critical roles in mediating polymerase switching during TLS, we tested whether PCNA binding by TFII-I was required for TLS. We created a TFII-I mutant with all four APIM motifs mutated to alanine (TFII-I mAPIM). The endogenous TFII-I interacted with PCNA (Figure 6B). Myc-TFII-I wild type (WT), but not the Myc-TFII-I mAPIM mutant protein, interacted with PCNA in cells depleted of endogenous TFII-I (Figure 6B). Moreover, consistent with an earlier report [20], GFP-TFII-I WT, but not GFP-TFII-I mAPIM, was recruited to laser-induced DNA damage sites in U2OS cells, along with DsRed-PCNA (Figure 6C). This result confirmed that TFII-I mAPIM lost its functional interaction with PCNA. Importantly, Myc-TFII-I mAPIM still interacted with Rev7 (Figure 6B). Compared to Myc-TFII-I WT, the Myc-TFII-I mAPIM mutant protein was significantly less efficient in rescuing TLS defects caused by TFII-I RNAi (Figure 6D). Thus, the PCNA-binding activity of TFII-I is required for its function in TLS.

The simplest model to explain the involvement of TFII-I in TLS is that TFII-I binds simultaneously to both PCNA and Rev7, bridging an interaction between the two proteins and contributing to the recruitment of Pol ζ to DNA lesions. Unfortunately, we could not detect the recruitment of GFP-Rev7 to laser-induced DNA damage sites, barring us from testing this notion using cytological methods. We, therefore, tested this hypothesis using IP-Western methods. Interactions among PCNA, TFII-I, and Rev7 were detectable in unirradiated cell lysates (Figure S5A). These interactions were enhanced following UV irradiation. More importantly, depletion of TFII-I abolished the interaction between PCNA and Rev7 in both cases (Figure S5A). Expression of Myc-TFII-I WT, but not mAPIM, restored the interaction between PCNA and Rev7 (Figure 6B). These results suggest that TFII-I bridges an interaction between PCNA and Rev7 in human cells, and that this function of TFII-I requires its APIM motifs.

### TFII-I dimerization is required for TLS and bridges the Rev7–PCNA interaction

**Figure 7:**
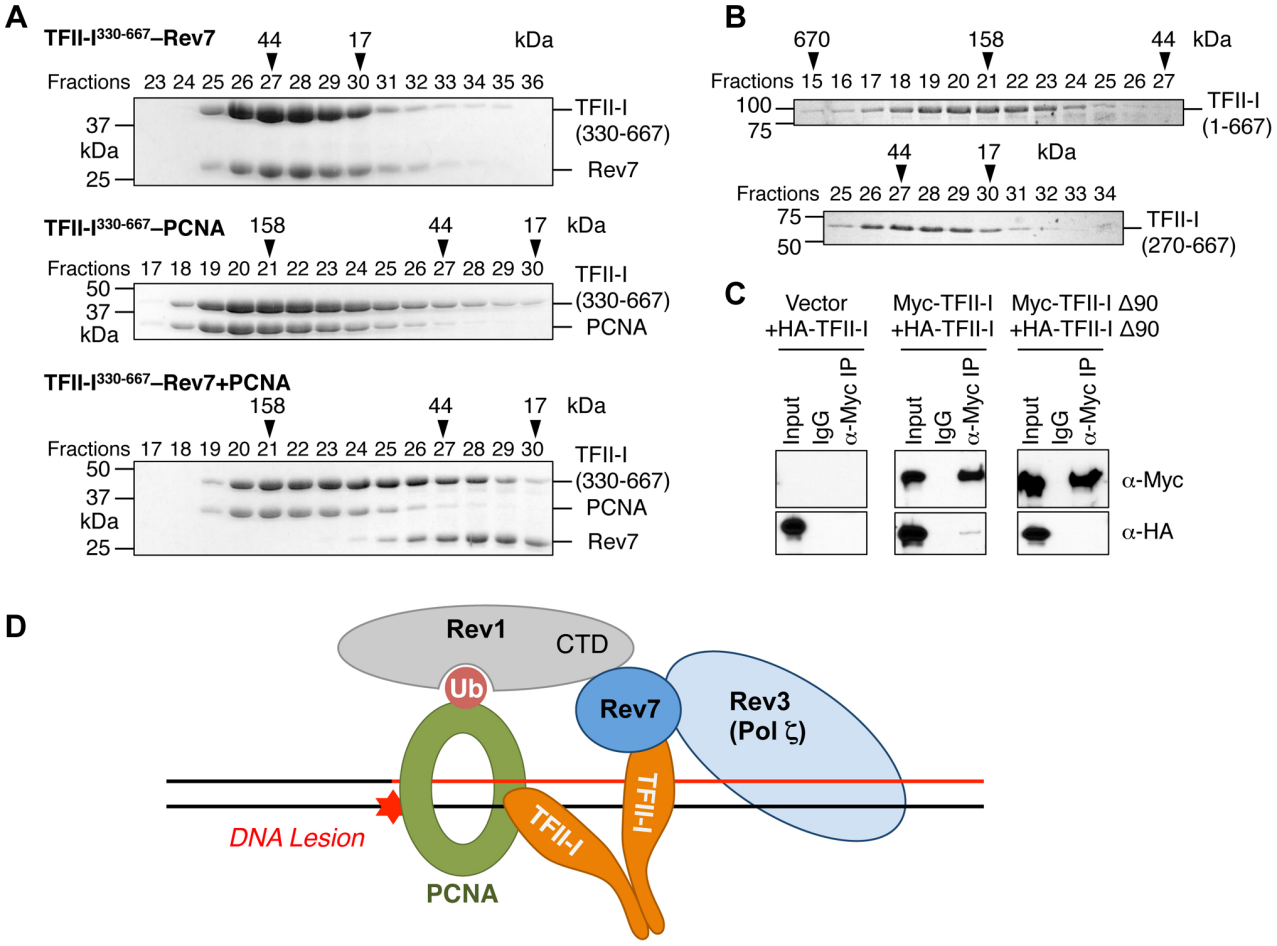
The TFII-I dimer bridges PCNA and Rev7. (A) The monomeric TFII-I fragment (residues 330–667) does not form a ternary complex with PCNA and Rev7. The indicated combinations of recombinant purified Rev7 R124A, PCNA, and TFII-I (residues 330–667) proteins were fractionated on a Superdex 200 gel filtration column. Selected fractions were separated by SDS-PAGE followed by Coomassie staining. The positions of native molecular mass standards are indicated by arrowheads. (B) Purified TFII-I^1–667^ and TFII-I^270–667^ fragments were fractionated on a Superdex 200 gel filtration column. (C) HeLa Tet-On cells were transfected with the indicated plasmids. Lysates, IgG IP, and anti-Myc IP of these cells were blotted with the indicated antibodies. (D) Model for TFII-I-dependent recruitment of Pol ζ (Rev3L–Rev7) to PCNA at DNA damage sites during translesion synthesis. Ub, ubiquitin. CTD, C-terminal domain.

We next checked whether the recombinant purified TFII-I^330–667^ fragment could form a ternary complex with Rev7 and PCNA using gel filtration. To our surprise, we found that TFII-I^330–667^, Rev7, and PCNA did not form a ternary complex (Figure 7A). Addition of PCNA to the pre-formed TFII-I^330–667^–Rev7 complex produced a TFII-I^330–667^–PCNA binary complex and free Rev7. Thus, binding of PCNA and binding of Rev7 to a monomeric fragment of TFII-I are mutually exclusive.

On the other hand, TFII-I is known to homodimerize, and contains an N-terminal dimerization domain [19]. Indeed, a TFII-I fragment containing residues 1–667 fractionated with an apparent molecular mass of about 160 kD on gel filtration columns, which was consistent with it forming a homodimer (Figure 7B). By contrast, the TFII-I^270–667^ fragment that lacked the N-terminal dimerization domain fractionated as a monomer by gel filtration. Moreover, differentially tagged TFII-I, but not a TFII-I mutant protein lacking the first 90 residues (TFII-I Δ90), interacted with each other in human cells (Figure 7C), confirming that TFII-I could oligomerize *in vivo*. Finally, the monomeric TFII-I Δ90 mutant protein was defective in supporting TLS (see Figure 6D above). Consistently, this mutant could not restore the PCNA-Rev7 interaction in TFII-I-depleted cells (Figure 6B). Therefore, homodimerization of TFII-I is required for its function in TLS and for bridging the PCNA–Rev7 interaction. We propose that one monomer of a TFII-I dimer can bind to PCNA while the other can bind to Rev7 (Figure 7D). In this way, the TFII-I dimer bridges the interaction between PCNA and Rev7, and contributes to the recruitment of Pol ζ to DNA lesions during TLS.

Because Rev7 also interacts with the C-terminal domain (CTD) of Rev1, we tested whether recruitment of Rev1 to DNA damage sites was dependent on TFII-I or Rev7. GFP-Rev1 was recruited to laser-induced DNA damage sites in human cells (Figure S5B). Depletion of TFII-I or Rev7 did not alter this recruitment. Thus, Rev1 is recruited to DNA damage sites independently of TFII-I and Rev7. Taken together, our results suggest that TFII-I and Rev1 collaborate to recruit Pol ζ to DNA damage sites through TFII-I–Rev7 and Rev1-CTD–Rev7 interactions (Figure 7D).

## Discussion

Pol ζ plays critical roles in DNA translesion synthesis (TLS), cellular DNA damage tolerance, and the maintenance of genomic stability. In this study, we have discovered the transcription factor TFII-I as a new, functionally important interactor of Pol ζ in human cells. We found that PCNA binding and dimerization of TFII-I are required for efficient TLS. Our study thus provides key insights into the mechanism and regulation of Pol ζ in human cells.

We propose the following model to explain the involvement of TFII-I in TLS (Figure 7D). In this model, Rev1 and the TFII-I homodimer are independently recruited to ubiquitinated PCNA at DNA damage sites. This complex then simultaneously engages Rev7 and recruits Pol ζ to these lesions. Rev1 also anchors Pol η, ι, or κ to PCNA. After these Y-family polymerases insert nucleotides directly opposite to the DNA lesion, Pol ζ extends DNA synthesis past the lesion. Because TFII-I specifically interacts with the Pol ζ subunit Rev7, but not with Pol η or ι, we speculate that TFII-I might also mediate polymerase switching from Pol η/ι/κ to Pol ζ.

In support of a role of TFII-I in recruiting Pol ζ to DNA lesions, we showed that TFII-I bridges an interaction between PCNA and Rev7 in UV-irradiated human cells, using IP-Western experiments. We could not reconstitute a complex containing TFII-I, PCNA, Rev7, Rev3, and Rev1 *in vitro* using purified recombinant proteins, due to the difficulty of expressing full-length TFII-I and larger fragments of Rev3 and Rev1. Complex formation might also require DNA or additional accessory subunits of Pol ζ [39]-[41].

Rev3L has been reported to contain a putative APIM motif [20]. In addition, PolD3 (p66), an accessory subunits of Pol ζ, contains a functional PCNA-binding PIP motif [41], [42]. Furthermore, in addition to its ability to bind ubiquitin on ubiquitinated PCNA, Rev1 has been implicated in direct binding to unmodified PCNA [8], [43], [44]. Therefore, along with our finding that TFII-I binds to PCNA and Rev7, it is clear that the TLS machinery makes multiple contacts with PCNA. A cell-free system that can support PCNA- and Pol ζ-dependent TLS is needed to definitively establish the role of TFII-I in this process and dissect the relative contributions of the multiple PCNA-binding mechanisms. Finally, there are no known TFII-I orthologs in the budding yeast. It is possible that yeast Pol ζ uses distinct mechanisms to interact with PCNA.

We were unable to directly test whether TFII-I is required for Pol ζ recruitment to DNA damage sites, as we could not detect the enrichment of either endogenous Rev7 at UV-induced nuclear foci using immunofluorescence or the recruitment of GFP-Rev7 to laser-induced DNA damage sites. The underlying reason for the lack of Rev7 enrichment at DNA damage sites is unclear, but could be due to the transient nature of the TFII-I/Rev1-bridged interactions between PCNA and Pol ζ. Alternatively, the Rev1–Rev7 and TFII-I–Rev7 interactions are required, but are not sufficient, to recruit Rev7 to the site of DNA damage. Only the intact, functional Pol ζ (i.e. the Rev3L–Rev7 complex) can be efficiently recruited. Because Rev3L is a low-abundance protein in human cells, recruitment of Pol ζ to DNA damage sites might be below the detection limits of our cytological assays.

Two lines of evidence suggest that the TLS function of TFII-I is independent of its roles in transcription. First, depletion of TFII-I causes only a mild transcription defect in human cells. Of the few genes whose expression was down-regulated by TFII-I depletion, none had known roles in TLS. Second, the PCNA-binding APIM motifs of TFII-I are critical for TLS. These motifs do not have expected roles in transcription.

Williams-Beuren syndrome (WBS) is a rare genetic disorder caused by deletion of one copy of the chromosome 7q11.23 region, which contains TFII-I and about 25 other genes [22]. WBS patients have multiple symptoms, including distinctive craniofacial features, mild mental retardation, and cardiovascular defects. Different phenotypes have been linked to different genes in the 7q11.23 region. Mice with a heterozygous deletion of N-terminal 140 residues of TFII-I exhibit craniofacial and neurobehavioral alterations [23], implicating this region of TFII-I in WBS pathophysiology. In this study, we showed that the N-terminal region of TFII-I is critical for TLS, raising the intriguing possibility that defective TLS might underlie a subset of symptoms in WBS. It will be interesting to test whether cells derived from WBS patients exhibit sensitivity to UV irradiation and are defective in TLS, and may have defects in components of this TLS complex (Figure 7D).

In addition to TLS, Pol ζ is involved in somatic hypermutation, DNA interstrand crosslink repair, and DSB repair through homologous recombination [35], [45]. Future experiments are needed to test whether TFII-I also contributes to the functions of Pol ζ in these processes. Furthermore, inactivation of Pol ζ sensitizes human cancer cells to killing by the chemotherapeutic drug, cisplatin [32]. Chemical compounds targeting Pol ζ may enhance the efficacy of cisplatin. Our discovery of TFII-I as a novel Pol ζ regulatory factor presents new opportunities for the chemical inhibition of this important polymerase complex. Finally, the general transcription factor TFIIH has a well-established role in nucleotide excision repair [1]. Our findings linking TFII-I to TLS further strengthen the general principle of component sharing in diverse nuclear processes.

## Materials and Methods

### Cell culture, transfection, and UV irradiation

HeLa Tet-On, 293T, and U2OS cells were grown in Dulbecco's modified Eagle's medium (DMEM; Invitrogen) supplemented with 10% fetal bovine serum (FBS). Plasmid and siRNA transfections were performed with the Effectene reagent (Qiagen), Lipofectamine 2000, and Lipofectamine RNAiMAX (Invitrogen) for 48 hrs in the indicated cell lines before the desired analysis unless otherwise noted. To establish the TAP-Rev7 cell line, 293T cells were transfected with the pIRES-Puro-TAP (Clontech) or pIRES-Puro-TAP-Rev7 vectors, and selected with 2 µg/ml puromycin. Individual clones were isolated for further analysis. The following siRNAs were chemically synthesized at or purchased from Dharmacon: siControl (5′-GACCGUUAGGUACAGAAGAUU-3′), siLuc, (5′-UCAUUCCGGAUACUGCGAU-3′), siRev7-1 (5′-CGGACAUUUUAAAGAUGCA-3), siRev7-2 (5′UGCAUCUUUAAAAUGUCCG-3′), and siGENOME Smartpools against human Rev3L and TFII-I. For UV-C (254 nm) treatment, the growth medium was removed from the cells and reserved. Cells were washed twice with PBS. The plates (without PBS) were transferred to a UV cross-linker (Stratagene) and irradiated with the indicated UV doses. The UV-C dose delivered to the cells was confirmed with a UV radiometer (UVP, Inc.). The reserved medium was added back to cells. The cells were returned to the incubator.

### Tandem affinity purification

For tandem affinity purification of TAP-Rev7, ten 150-mm dishes of 293T cells stably expressing TAP-Rev7 were harvested in the TAP lysis buffer (50 mM HEPES pH 7.5, 100 mM KCl, 2 mM EDTA, 10% glycerol, 0.1% NP-40, 10 mM NaF, 0.25 mM Na_3_VO_4_, 50 mM β-glycerolphosphate, 2 mM DTT, and 1X protease inhibitor cocktail). Cleared lysates were bound to IgG-Sepharose beads (GE Amersham) for 4 hrs at 4°C. Beads were subsequently washed three times with the lysis buffer and once with the TEV buffer (10 mM HEPES pH 8.0, 150 mM NaCl, 0.1% NP-40, 0.5 mM EDTA, 1 mM DTT, and 1X protease inhibitor cocktail). Protein complexes were cleaved off the beads by 70 µg TEV protease in TEV buffer overnight at 4°C. Supernatants were diluted in calmodulin-binding buffer (10 mM HEPES pH 8.0, 150 mM NaCl, 1 mM magnesium acetate, 1 mM imidazole, 0.1% NP-40, 6 mM CaCl_2_, 10 mM 2- mercaptoethanol) and incubated with calmodulin-sepharose beads (GE Amersham) for 90 minutes at 4°C. Captured protein complexes were washed three times with the calmodulin-binding buffer and the calmodulin rinse buffer (50 mM NH_4_HCO_3_ pH 8.0, 75 mM NaCl, 1 mM magnesium acetate, 1 mM imidazole, 2 mM CaCl_2_). Proteins were eluted in SDS sample buffer, boiled for 10 min, concentrated in microcon concentrators (Millipore), and subjected to SDS-PAGE. Gels were stained with colloidal Coomassie blue stain (Pierce) according to manufacturer's protocols. Unique bands were excised and in-gel proteolysis was performed using modified porcine trypsin digestion overnight. The resulting peptide mixture was dissolved and subjected to nano-LC/MS/MS analysis on a ThermoFinnigan LTQ instrument, coupled with an Agilent 1100 Series HPLC system. Peptide sequences were identified using the Mascot search engine (Matrix science). Those proteins identified in the TAP-REV7 purification with multiple peptides and not identified in the TAP-vector control pull-downs were considered hits.

### Antibodies, immunoblotting, immunoprecipitation, and immunofluorescence

The antibodies used in this study are: α-Myc (Roche), α-Rev7 (BD Transduction), α-TFII-I (Bethyl, A301-330A), α-Pol ι (Bethyl, A301-303A), α-Pol η (Abcam, ab17725), α-tubulin (Sigma), α-γH2AX (Millipore, 05-636), and α-PCNA (Santa Cruz, PC10). For immunoblotting and immunofluorescence, the antibodies were used at a final concentration of 1 µg/ml.

For immunoblotting, cells were lysed in SDS sample buffer, sonicated, boiled, separated by SDS–PAGE, and blotted with the indicated antibodies. Horseradish peroxidase-conjugated goat anti-rabbit or anti-mouse IgG (Amersham Biosciences) was used as the secondary antibodies. Immunoblots were developed using the ECL reagent (Amersham Biosciences) according to the manufacturer's protocols and exposed to film.

For immunoprecipitation, cells were lysed with the lysis buffer (50 mM Tris-HCl, pH 8.0, 250 mM NaCl, 5 mM MgCl_2_, 5 mM EDTA, 0.5% Triton X-100, 10 mM NaF, 80 mM β-glycerophosphate, 10% glycerol, 1 mM DTT, and protease inhibitor cocktail). The lysates were cleared by centrifugation for 30 min at 4°C at top speed in a microcentrifuge. Control IgG (Sigma) or α-TFII-I antibodies were covalently coupled to Affi-Prep protein A beads (Bio-Rad). The supernatants were incubated the antibody-coupled beads. The beads were washed with the lysis buffer. Proteins bound to the beads were dissolved in SDS sample buffer, boiled, separated by SDS-PAGE, and blotted with α-Rev7 and α-TFII-I antibodies.

For the immunoprecipitation of the PCNA complex, U2OS cells were fixed in PBS containing 0.25% formaldehyde for 10 min at room temperature, and the reaction was stopped by the addition of glycine to a final concentration of 0.125 M. After being washed twice with PBS, cells were resuspended in Lysis Buffer 1 (10 mM HEPES pH 6.5, 10 mM EDTA, 0.5 mM EGTA, 0.25% Triton X-100, 1X protease inhibitor) and kept on ice for 10 min. Following centrifugation at 1700 g for 10 min at 4°C, pellets were washed with Lysis Buffer 2 (10 mM HEPES pH 6.5, 200 mM NaCl, 10 mM EDTA, 0.5 mM EGTA, 1X protease inhibitor) and again pelleted at 1700 g for 5 min at 4°C. Pellets were then resuspended in Lysis Buffer 3 (25 mM Tris-HCl pH 6.5, 100 mM NaCl, 2 mM MgCl_2_, 1X protease inhibitor, 10% glycerol, 1 mM DTT, 10 mM BGP, 5 mM NaF, 3 mM NaVO_4_, Turbo nuclease), incubated on ice for 10 min, and sonicated. Lysates were then centrifuged at 14,000 rpm for 15 min at 4°C. The supernatant was incubated with Affi-Prep Protein A beads coupled to α-PCNA for 3 h at 4°C. Beads were washed five times with Lysis Buffer 3. Protein crosslinks were reversed by incubating the beads in SDS buffer at 95°C for 30 min. Proteins bound to beads were analyzed by SDS-PAGE and immunoblotting.

For immunofluorescence, HeLa Tet-On cells transfected with the indicated siRNAs were plated in four-well chamber slides (LabTek), treated with 10 J/m^2^ UV or left untreated, and fixed with 4% paraformaldehyde in 250 mM HEPES pH 7.4, 0.1% Triton X-100 at 4°C for 20 min. After 3–5 washes over 20 min in PBS, cells were permeabilized in PBS containing 0.5% Triton X-100 for 20 min, and then washed with PBS. The cells were blocked in PBS containing 5% BSA followed by a 2-h incubation with the primary antibodies. After 3–5 washes over 20 min with PBS, cells were incubated with fluorescent secondary antibodies (Alexa Fluor 488 or 647, Molecular Probes) for 30 min at room temperature. After incubation, cells were washed with PBS, and their nuclei were stained with DAPI (1 µg/ml). Slides were mounted and viewed with a 100X objective on a DeltaVision microscope. All images were taken at 0.2 µm intervals, deconvolved, and stacked. The images were further processed in ImageJ.

### *In vitro* protein binding assay

For GST pulldown assays, Myc-TFII-I or its fragments were *in vitro* translated in rabbit reticulocyte lysate in the presence of ^35^S-methionine and incubated with bacterially expressed GST or GST-Rev7 in the binding buffer (25 mM Tris-HCl pH 8.0, 2.7 mM KCl, 137 mM NaCl, 0.05% Tween-20) for 1 h at room temperature. Protein complexes were then bound to Glutathione-Sepharose beads for 30 min at room temperature. After 5 washes with the binding buffer, the proteins were eluted with SDS sample buffer, boiled, and subjected to SDS-PAGE. The bound proteins were analyzed with a phosphor imager (Fujifilm) to visualize ^35^S-labeled TFII-I and Coomassie staining to visualize GST and GST-Rev7.

### Expression and purification of human Rev7-Rev3L, PCNA, TFII-I, and Rev1

Human His_6_-Rev7 R124A mutant bound to the Rev7-binding region of human Rev3L (residues 1847–1898) and untagged human PCNA were prepared as previously described [46], [47]. (Rev7 forms dimers in vitro, but in vivo function of this dimerization event is unclear. The R124A mutation disrupts Rev7 dimerization.) Human TFII-I fragments and the C-terminal domain (CTD) of Rev1 (residues 1140–1251) were expressed as GST-fusion proteins in bacteria and purified with the glutathione-Sepharose 4B resin (GE Healthcare). The eluted proteins were digested with the PreScission protease (GE Healthcare), and further purified with anion exchange and size exclusion chromatography. To assay for complex formation and to determine the apparent molecular weight of the complexes, the gel filtration standard (Bio-Rad, 151-1901), Rev7–Rev3L–TFII-I, PCNA–TFII-I, Rev7–Rev3L–Rev1–TFII-I, and Rev7–Rev3L–TFII-I in the presence of PCNA were fractionated on a Superdex 200 10/300 GL column (GE Healthcare).

### Colony formation assay

293T cells were transfected twice with the indicated siRNAs in a 24 h period, and replated into six-well plates at 60 h after the first siRNA treatment, with 500, 2000, 10,000, and 40,000 cells per well. After another 24 h, cells were exposed to varying doses of UV (0, 4, 8, 12, and 16 J/m^2^). Twelve days later, colonies were fixed and stained in a solution containing 3∶1 methanol and glacial acetic acid plus 1% trypan blue (Sigma). Colonies containing 50 or more cells were counted. The surviving fractions for each group represent the plating efficiency for each treatment divided by the plating efficiency of the corresponding untreated control samples.

### Mutation frequency assay

293T cells were transfected with the appropriate siRNAs. At 24 h after siRNA transfection, pSP189 plasmids were irradiated with UV (1000 J/m^2^) and transfected into the cells using Lipofectamine 2000 (Invitrogen). Cells were harvested 48 h later for plasmid purification using the DNA miniprep kit (QIAGEN). The purified plasmids were digested with DpnI and transformed into the bacterial strain MBM7070 by electroporation. Bacterial cells with wild-type *SupF* tRNA expressed functional β-galactosidase and formed blue colonies on X-gal plates, whereas bacteria with mutated *SupF* formed white colonies. The mutation frequency in the *SupF* gene was analyzed by counting the ratio between blue (wild-type) and white (mutant) colonies. Mutations in the *SupF* gene were confirmed by DNA sequencing.

### Flow cytometry

HeLa Tet-On cells transfected with the appropriate siRNAs were irradiated with 10 J/m^2^ UV. Samples were taken at the indicated timepoints, fixed with 70% ice-cold ethanol, blocked with PBS containing 5% BSA and 0.25% Triton-X100, and stained with the anti-γ-H2AX monoclonal antibody. Cells were washed, incubated with the Alexa Fluor 488 donkey anti-mouse secondary antibody (Invitrogen), and counterstained with propidium iodide in PBS containing RNase A. Cells were analyzed with a BD FACSCalibur flow cytometer by using the CellQuest software. Data were processed with FlowJo (FloJo, Ashland, OR).

### Microarray and quantitative PCR

Total RNA was harvested from untreated and siRNA-treated HeLa Tet-On cells at 48 h after siRNA transfection using the RNeasy Kit (Qiagen). cDNA was synthesized from the total RNA, purified, and hybridized to a HumanHT-12 v4 BeadChip array at the UTSW Microarray Core facility. The arrays were then washed, stained, and scanned according to the manufacturer's protocol. For quantitative PCR (qPCR), cells were lysed in Trizol (Invitrogen). Total RNA was extracted by chloroform extraction and isopropanol precipitation. About 1–2 µg of total RNA was reverse transcribed with the high-capacity cDNA reverse transcription kit (Applied Biosystems) according to the manufacturer's instructions. Taqman probes for human Rev3L (Hs01076848_m1), Rev1 (Hs01019771_m1), Rev7 (Hs01057448_m1), and Rad18 (Hs00892551_m1), and GAPDH (Applied Biosystems) were used for qPCR in TaqMan master mix (Applied Biosystems) according to the manufacturer's protocol. Samples were run in triplicates with the appropriate negative controls.

### Laser micro-irradiation

U2OS cells were transfected with DsRed-PCNA and GFP-TFII-I or GFP-Rev1 along with the appropriate siRNAs. DSBs were introduced in the nuclei of cultured cells by microirradiation with a pulsed nitrogen laser (Spectra-Physics; 365 nm, 10 Hz pulse) [48]. The laser system was directly coupled (Micropoint Ablation Laser System; Photonic Instruments, Inc.) to the epifluorescence path of an Axiovert 200 M microscope (Carl Zeiss MicroImaging, Inc.) for immunostaining imaging or time-lapse imaging and focused through a Plan-Apochromat 63×/NA 1.40 oil immersion objective (Carl Zeiss MicroImaging, Inc.). The output of the laser power was set at 60% of the maximum. Time-lapse images were taken with an AxioCam HRm (Carl Zeiss MicroImaging, Inc.). During microirradiation, imaging, or analysis, the cells were maintained at 37°C in 35-mm glass-bottom culture dishes (MatTek Cultureware). The growth medium was replaced by CO_2_-independent medium (Invitrogen) before analysis. The images were further processed by ImageJ and Photoshop.

## Supporting Information

Figure S1 TFII-I interacts with Rev in human cells and *in vitro*. (A) U2OS cells were irradiated with UV (60 J/m^2^) and treated with formaldehyde. Lysates, IgG IP, and anti-TFII-I IP of these cells were blotted with the indicated antibodies. (B, C) The indicated ^35^S-TFII-I fragments in rabbit reticulocyte lysate (Input) or bound to GST or GST-Rev7 beads were separated by SDS-PAGE and analyzed with a phosphor imager (top panel) or stained with Coomassie (bottom panel).
(JPG)

Figure S2 TFII-I forms a complex with Rev7, the Rev7-binding region of Rev3, and the C-terminal domain (CTD) of Rev1. (A) Ribbon drawing of the structure of the Rev7–Rev3–Rev1–Pol κ complex (PDB code, 4FJO). The seat belt-like structural element of closed Rev7 is colored green. (B) Purified recombinant TFII-I (residues 350-667), the Rev7 R124A–Rev3L (residues 1847-1898) complex, and Rev1 CTD were mixed at 1∶1∶1 molar ratios and fractioned on a Superdex 200 column. The indicated column fractions were separated by SDS-PAGE and stained with Coomassie. A degradation product of TFII-I was labeled with an asterisk. The eluting positions of the native molecular mass standards are indicated by arrowheads.
(JPG)

Figure S3 **Rev3L and Rev7 are required for DNA damage tolerance in human cells.** HeLa Tet-On cells were transfected with the indicated siRNAs, treated with UV (10 J/m^2^), and harvested at various timepoints for flow cytometry. Representative dot plots at the selected timepoints are shown. The percentages of γ-H2AX-positive cells are shown at the upper right corner of each plot.
(JPG)

Figure S4 Depletion of TFII-I or Pol ζ does not alter the TLS mutation spectrum. (A-D) Position and type of mutations in the UV-irradiated *SupF* gene recovered from 293T cells transfected with the indicated siRNAs.
(JPG)

Figure S5 TFII-I is required for the PCNA–Rev7 interaction, but is dispensable for Rev1 recruitment to laser-induced DNA damage sites. (A) U2OS cells were mock transfected or transfected with siTFII-I, left untreated or irradiated with UV (60 J/m^2^), and treated with formaldehyde. Lysates and anti-PCNA IP of these cells were blotted with the indicated antibodies. (B) U2OS cells were transfected with GFP-Rev1 and DsRed-PCNA and the indicated siRNAs, and micro-irradiated with a 365-nm laser along straight lines. The GFP and DsRed channels are shown separately in gray scale and together in the merge. Scale bar, 10 µm.
(JPG)

Table S1 Mass spectrometry analysis of TAP-Rev7 binding proteins.
(PDF)

## References

[1] Sancar A, Lindsey-Boltz LA, Unsal-Kacmaz K, Linn S (2004) Molecular mechanisms of mammalian DNA repair and the DNA damage checkpoints. Annu Rev Biochem 73: 39–85. 15189136 10.1146/annurev.biochem.73.011303.073723

[2] Sale JE, Lehmann AR, Woodgate R (2012) Y-family DNA polymerases and their role in tolerance of cellular DNA damage. Nat Rev Mol Cell Biol 13: 141–152. 22358330 10.1038/nrm3289 PMC3630503

[3] Zan H, Komori A, Li Z, Cerutti A, Schaffer A, et al (2001) The translesion DNA polymerase zeta plays a major role in Ig and bcl-6 somatic hypermutation. Immunity 14: 643–653. 11371365 10.1016/s1074-7613(01)00142-x PMC4632985

[4] Zan H, Shima N, Xu Z, Al-Qahtani A, Evinger Iii AJ, et al (2005) The translesion DNA polymerase theta plays a dominant role in immunoglobulin gene somatic hypermutation. EMBO J 24: 3757–3769. 16222339 10.1038/sj.emboj.7600833 PMC1276717

[5] Saribasak H, Maul RW, Cao Z, Yang WW, Schenten D, et al (2012) DNA polymerase zeta generates tandem mutations in immunoglobulin variable regions. J Exp Med 209: 1075–1081. 22615128 10.1084/jem.20112234 PMC3371727

[6] Bergink S, Jentsch S (2009) Principles of ubiquitin and SUMO modifications in DNA repair. Nature 458: 461–467. 19325626 10.1038/nature07963

[7] Garg P, Burgers PM (2005) Ubiquitinated proliferating cell nuclear antigen activates translesion DNA polymerases eta and REV1. Proc Natl Acad Sci U S A 102: 18361–18366. 16344468 10.1073/pnas.0505949102 PMC1317920

[8] Wood A, Garg P, Burgers PM (2007) A ubiquitin-binding motif in the translesion DNA polymerase Rev1 mediates its essential functional interaction with ubiquitinated proliferating cell nuclear antigen in response to DNA damage. J Biol Chem 282: 20256–20263. 17517887 10.1074/jbc.M702366200

[9] Murakumo Y, Ogura Y, Ishii H, Numata S, Ichihara M, et al (2001) Interactions in the error-prone postreplication repair proteins hREV1, hREV3, and hREV7. J Biol Chem 276: 35644–35651. 11485998 10.1074/jbc.M102051200

[10] Guo C, Fischhaber PL, Luk-Paszyc MJ, Masuda Y, Zhou J, et al (2003) Mouse Rev1 protein interacts with multiple DNA polymerases involved in translesion DNA synthesis. EMBO J 22: 6621–6630. 14657033 10.1093/emboj/cdg626 PMC291821

[11] Ross AL, Simpson LJ, Sale JE (2005) Vertebrate DNA damage tolerance requires the C-terminus but not BRCT or transferase domains of REV1. Nucleic Acids Res 33: 1280–1289. 15741181 10.1093/nar/gki279 PMC552965

[12] Ohashi E, Murakumo Y, Kanjo N, Akagi J, Masutani C, et al (2004) Interaction of hREV1 with three human Y-family DNA polymerases. Genes Cells 9: 523–531. 15189446 10.1111/j.1356-9597.2004.00747.x

[13] Kosarek JN, Woodruff RV, Rivera-Begeman A, Guo C, D'Souza S, et al (2008) Comparative analysis of in vivo interactions between Rev1 protein and other Y-family DNA polymerases in animals and yeasts. DNA Repair (Amst) 7: 439–451. 18242152 10.1016/j.dnarep.2007.11.016 PMC2363158

[14] Johnson RE, Washington MT, Haracska L, Prakash S, Prakash L (2000) Eukaryotic polymerases iota and zeta act sequentially to bypass DNA lesions. Nature 406: 1015–1019. 10984059 10.1038/35023030

[15] Shachar S, Ziv O, Avkin S, Adar S, Wittschieben J, et al (2009) Two-polymerase mechanisms dictate error-free and error-prone translesion DNA synthesis in mammals. EMBO J 28: 383–393. 19153606 10.1038/emboj.2008.281 PMC2646147

[16] Livneh Z, Ziv O, Shachar S (2010) Multiple two-polymerase mechanisms in mammalian translesion DNA synthesis. Cell Cycle 9: 729–735. 20139724 10.4161/cc.9.4.10727

[17] Wojtaszek J, Lee CJ, D'Souza S, Minesinger B, Kim H, et al (2012) Structural basis of Rev1-mediated assembly of a quaternary vertebrate translesion polymerase complex consisting of Rev1, heterodimeric polymerase (Pol) zeta, and Pol kappa. J Biol Chem 287: 33836–33846. 22859295 10.1074/jbc.M112.394841 PMC3460478

[18] Roy AL, Malik S, Meisterernst M, Roeder RG (1993) An alternative pathway for transcription initiation involving TFII-I. Nature 365: 355–359. 8377828 10.1038/365355a0

[19] Cheriyath V, Roy AL (2001) Structure-function analysis of TFII-I. Roles of the N-terminal end, basic region, and I-repeats. J Biol Chem 276: 8377–8383. 11113127 10.1074/jbc.M008411200

[20] Gilljam KM, Feyzi E, Aas PA, Sousa MM, Muller R, et al (2009) Identification of a novel, widespread, and functionally important PCNA-binding motif. J Cell Biol 186: 645–654. 19736315 10.1083/jcb.200903138 PMC2742182

[21] Roy AL (2012) Biochemistry and biology of the inducible multifunctional transcription factor TFII-I: 10 years later. Gene 492: 32–41. 22037610 10.1016/j.gene.2011.10.030 PMC3246126

[22] Pober BR (2010) Williams-Beuren syndrome. N Engl J Med 362: 239–252. 20089974 10.1056/NEJMra0903074

[23] Lucena J, Pezzi S, Aso E, Valero MC, Carreiro C, et al (2010) Essential role of the N-terminal region of TFII-I in viability and behavior. BMC Med Genet 11: 61. 20403157 10.1186/1471-2350-11-61 PMC2865459

[24] Luo X, Yu H (2008) Protein metamorphosis: the two-state behavior of Mad2. Structure 16: 1616–1625. 19000814 10.1016/j.str.2008.10.002 PMC2644451

[25] Aravind L, Koonin EV (1998) The HORMA domain: a common structural denominator in mitotic checkpoints, chromosome synapsis and DNA repair. Trends Biochem Sci 23: 284–286. 9757827 10.1016/s0968-0004(98)01257-2

[26] Luo X, Fang G, Coldiron M, Lin Y, Yu H, et al (2000) Structure of the Mad2 spindle assembly checkpoint protein and its interaction with Cdc20. Nat Struct Biol 7: 224–229. 10700282 10.1038/73338

[27] Luo X, Tang Z, Rizo J, Yu H (2002) The Mad2 spindle checkpoint protein undergoes similar major conformational changes upon binding to either Mad1 or Cdc20. Mol Cell 9: 59–71. 11804586 10.1016/s1097-2765(01)00435-x

[28] Luo X, Tang Z, Xia G, Wassmann K, Matsumoto T, et al (2004) The Mad2 spindle checkpoint protein has two distinct natively folded states. Nat Struct Mol Biol 11: 338–345. 15024386 10.1038/nsmb748

[29] Itoh G, Kanno S, Uchida KS, Chiba S, Sugino S, et al (2011) CAMP (C13orf8, ZNF828) is a novel regulator of kinetochore-microtubule attachment. EMBO J 30: 130–144. 21063390 10.1038/emboj.2010.276 PMC3020106

[30] Hara K, Hashimoto H, Murakumo Y, Kobayashi S, Kogame T, et al (2010) Crystal structure of human REV7 in complex with a human REV3 fragment and structural implication of the interaction between DNA polymerase zeta and REV1. J Biol Chem 285: 12299–12307. 20164194 10.1074/jbc.M109.092403 PMC2852969

[31] Kikuchi S, Hara K, Shimizu T, Sato M, Hashimoto H (2012) Structural basis of recruitment of DNA polymerase zeta by interaction between REV1 and REV7 proteins. J Biol Chem 287: 33847–33852. 22859296 10.1074/jbc.M112.396838 PMC3460479

[32] Cheung HW, Chun AC, Wang Q, Deng W, Hu L, et al (2006) Inactivation of human MAD2B in nasopharyngeal carcinoma cells leads to chemosensitization to DNA-damaging agents. Cancer Res 66: 4357–4367. 16618761 10.1158/0008-5472.CAN-05-3602

[33] McNally K, Neal JA, McManus TP, McCormick JJ, Maher VM (2008) hRev7, putative subunit of hPolzeta, plays a critical role in survival, induction of mutations, and progression through S-phase, of UV((254 nm))-irradiated human fibroblasts. DNA Repair (Amst) 7: 597–604. 18295554 10.1016/j.dnarep.2007.12.013 PMC4275125

[34] Jansen JG, Tsaalbi-Shtylik A, Hendriks G, Verspuy J, Gali H, et al (2009) Mammalian polymerase zeta is essential for post-replication repair of UV-induced DNA lesions. DNA Repair (Amst) 8: 1444–1451. 19783229 10.1016/j.dnarep.2009.09.006

[35] Sharma S, Hicks JK, Chute CL, Brennan JR, Ahn JY, et al (2012) REV1 and polymerase zeta facilitate homologous recombination repair. Nucleic Acids Res 40: 682–691. 21926160 10.1093/nar/gkr769 PMC3258153

[36] Parris CN, Seidman MM (1992) A signature element distinguishes sibling and independent mutations in a shuttle vector plasmid. Gene 117: 1–5. 1644298 10.1016/0378-1119(92)90482-5

[37] Chimge NO, Makeyev AV, Ruddle FH, Bayarsaihan D (2008) Identification of the TFII-I family target genes in the vertebrate genome. Proc Natl Acad Sci U S A 105: 9006–9010. 18579769 10.1073/pnas.0803051105 PMC2449355

[38] Ciccia A, Nimonkar AV, Hu Y, Hajdu I, Achar YJ, et al (2012) Polyubiquitinated PCNA recruits the ZRANB3 translocase to maintain genomic integrity after replication stress. Mol Cell 47: 396–409. 22704558 10.1016/j.molcel.2012.05.024 PMC3613862

[39] Makarova AV, Stodola JL, Burgers PM (2012) A four-subunit DNA polymerase zeta complex containing Pol delta accessory subunits is essential for PCNA-mediated mutagenesis. Nucleic Acids Res 40: 11618–11626. 23066099 10.1093/nar/gks948 PMC3526297

[40] Johnson RE, Prakash L, Prakash S (2012) Pol31 and Pol32 subunits of yeast DNA polymerase delta are also essential subunits of DNA polymerase zeta. Proc Natl Acad Sci U S A 109: 12455–12460. 22711820 10.1073/pnas.1206052109 PMC3411960

[41] Lee YS, Gregory MT, Yang W (2014) Human Pol zeta purified with accessory subunits is active in translesion DNA synthesis and complements Pol eta in cisplatin bypass. Proc Natl Acad Sci U S A 111: 2954–2959. 24449906 10.1073/pnas.1324001111 PMC3939873

[42] Bruning JB, Shamoo Y (2004) Structural and thermodynamic analysis of human PCNA with peptides derived from DNA polymerase-delta p66 subunit and flap endonuclease-1. Structure 12: 2209–2219. 15576034 10.1016/j.str.2004.09.018

[43] Guo C, Sonoda E, Tang TS, Parker JL, Bielen AB, et al (2006) REV1 protein interacts with PCNA: significance of the REV1 BRCT domain in vitro and in vivo. Mol Cell 23: 265–271. 16857592 10.1016/j.molcel.2006.05.038

[44] de Groote FH, Jansen JG, Masuda Y, Shah DM, Kamiya K, et al (2011) The Rev1 translesion synthesis polymerase has multiple distinct DNA binding modes. DNA Repair (Amst) 10: 915–925. 21752727 10.1016/j.dnarep.2011.04.033

[45] Ho TV, Guainazzi A, Derkunt SB, Enoiu M, Scharer OD (2011) Structure-dependent bypass of DNA interstrand crosslinks by translesion synthesis polymerases. Nucleic Acids Res 39: 7455–7464. 21666254 10.1093/nar/gkr448 PMC3177197

[46] Hara K, Shimizu T, Unzai S, Akashi S, Sato M, et al (2009) Purification, crystallization and initial X-ray diffraction study of human REV7 in complex with a REV3 fragment. Acta Crystallogr Sect F Struct Biol Cryst Commun 65: 1302–1305. 10.1107/S1744309109046181 PMC2802887 20054135

[47] Hishiki A, Shimizu T, Serizawa A, Ohmori H, Sato M, et al (2008) Crystallographic study of G178S mutant of human proliferating cell nuclear antigen. Acta Crystallogr Sect F Struct Biol Cryst Commun 64: 819–821. 10.1107/S174430910802277X PMC2531271 18765913

[48] Uematsu N, Weterings E, Yano K, Morotomi-Yano K, Jakob B, et al (2007) Autophosphorylation of DNA-PKCS regulates its dynamics at DNA double-strand breaks. J Cell Biol 177: 219–229. 17438073 10.1083/jcb.200608077 PMC2064131

