# Supplementary figures and images for: The Transcription Factor TFII-I Promotes DNA Translesion Synthesis and Genomic Stability
Source: PLoS Genet. 2014 Jun 12;10(6):e1004419. doi: 10.1371/journal.pgen.1004419 (PMC4055408; doi:10.1371/journal.pgen.1004419)

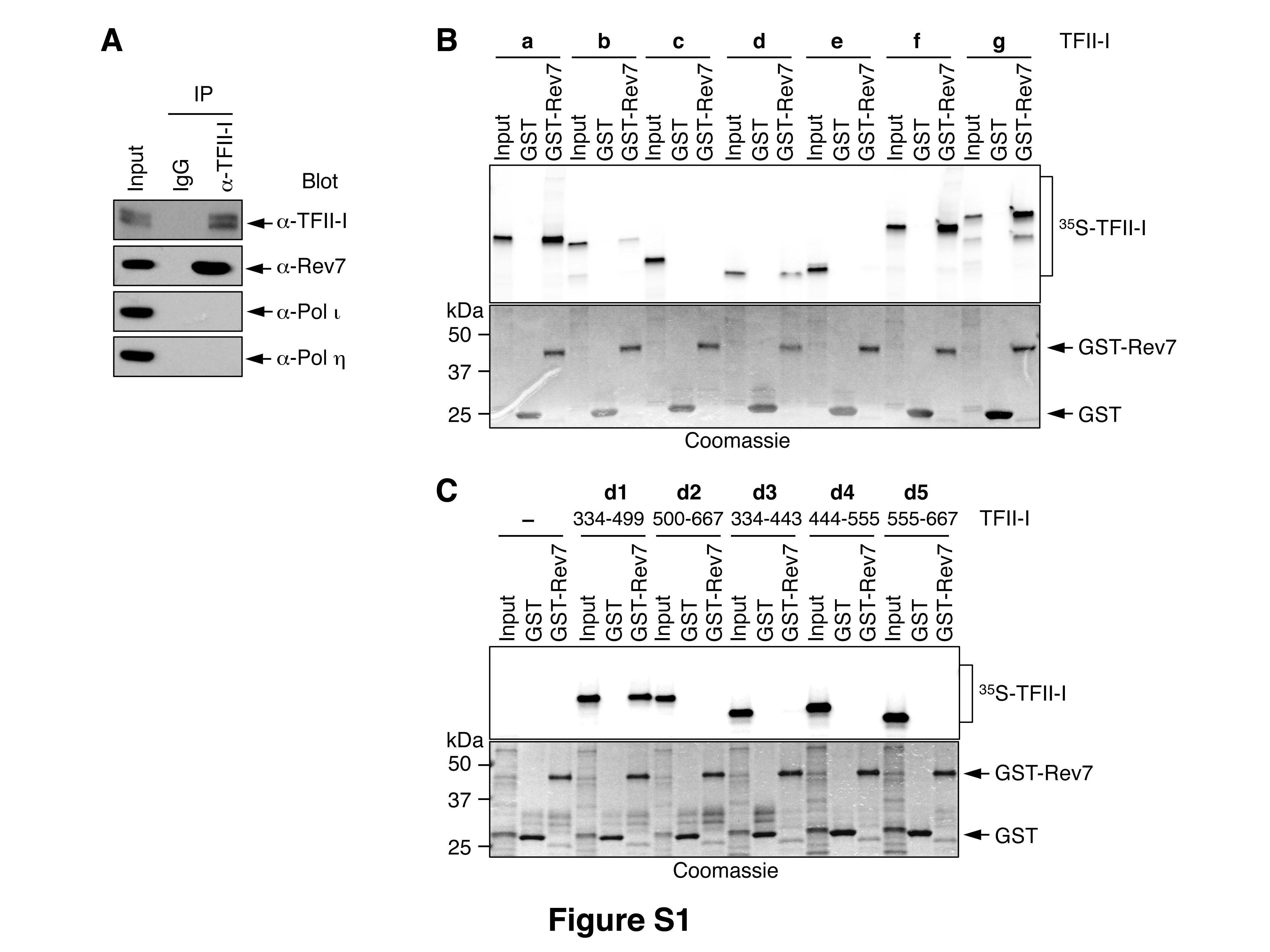

Supplement: Figure S1 — TFII-I interacts with Rev in human cells and in vitro. (A) U2OS cells were irradiated with UV (60 J/m2) and treated with formaldehyde. Lysates, IgG IP, and anti-TFII-I IP of these cells were blotted with the indicated antibodies. (B, C) The indicated 35S-TFII-I fragments in rabbit reticulocyte lysate (Input) or bound to GST or GST-Rev7 beads were separated by SDS-PAGE and analyzed with a phosphor imager (top panel) or stained with Coomassie (bottom panel). (JPG) [file pgen.1004419.s001.jpg]

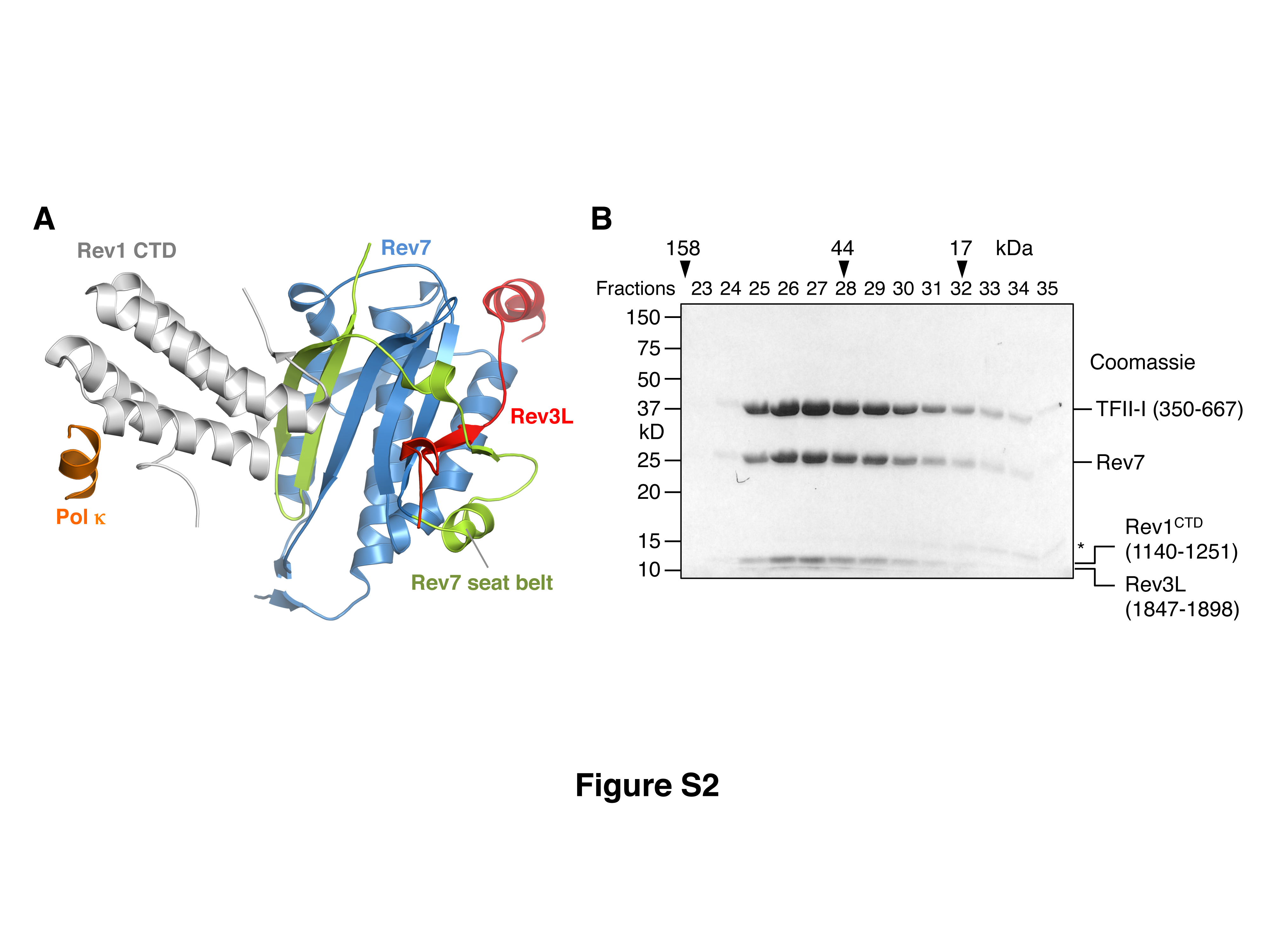

Supplement: Figure S2 — TFII-I forms a complex with Rev7, the Rev7-binding region of Rev3, and the C-terminal domain (CTD) of Rev1. (A) Ribbon drawing of the structure of the Rev7–Rev3–Rev1–Pol κ complex (PDB code, 4FJO). The seat belt-like structural element of closed Rev7 is colored green. (B) Purified recombinant TFII-I (residues 350-667), the Rev7 R124A–Rev3L (residues 1847-1898) complex, and Rev1 CTD were mixed at 1∶1∶1 molar ratios and fractioned on a Superdex 200 column. The indicated column fractions were separated by SDS-PAGE and stained with Coomassie. A degradation product of TFII-I was labeled with an asterisk. The eluting positions of the native molecular mass standards are indicated by arrowheads. (JPG) [file pgen.1004419.s002.jpg]

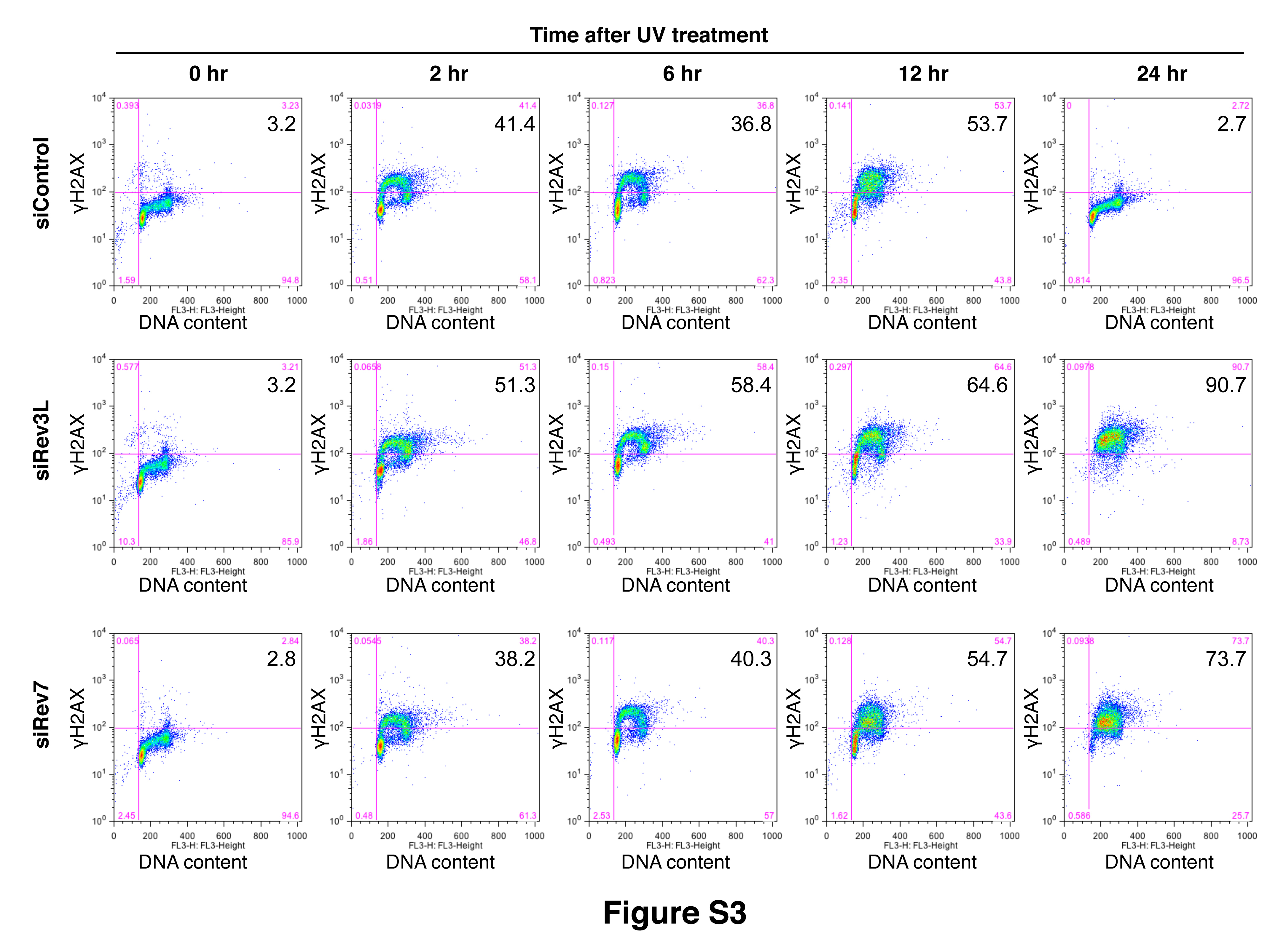

Supplement: Figure S3 — Rev3L and Rev7 are required for DNA damage tolerance in human cells. HeLa Tet-On cells were transfected with the indicated siRNAs, treated with UV (10 J/m2), and harvested at various timepoints for flow cytometry. Representative dot plots at the selected timepoints are shown. The percentages of γ-H2AX-positive cells are shown at the upper right corner of each plot. (JPG) [file pgen.1004419.s003.jpg]

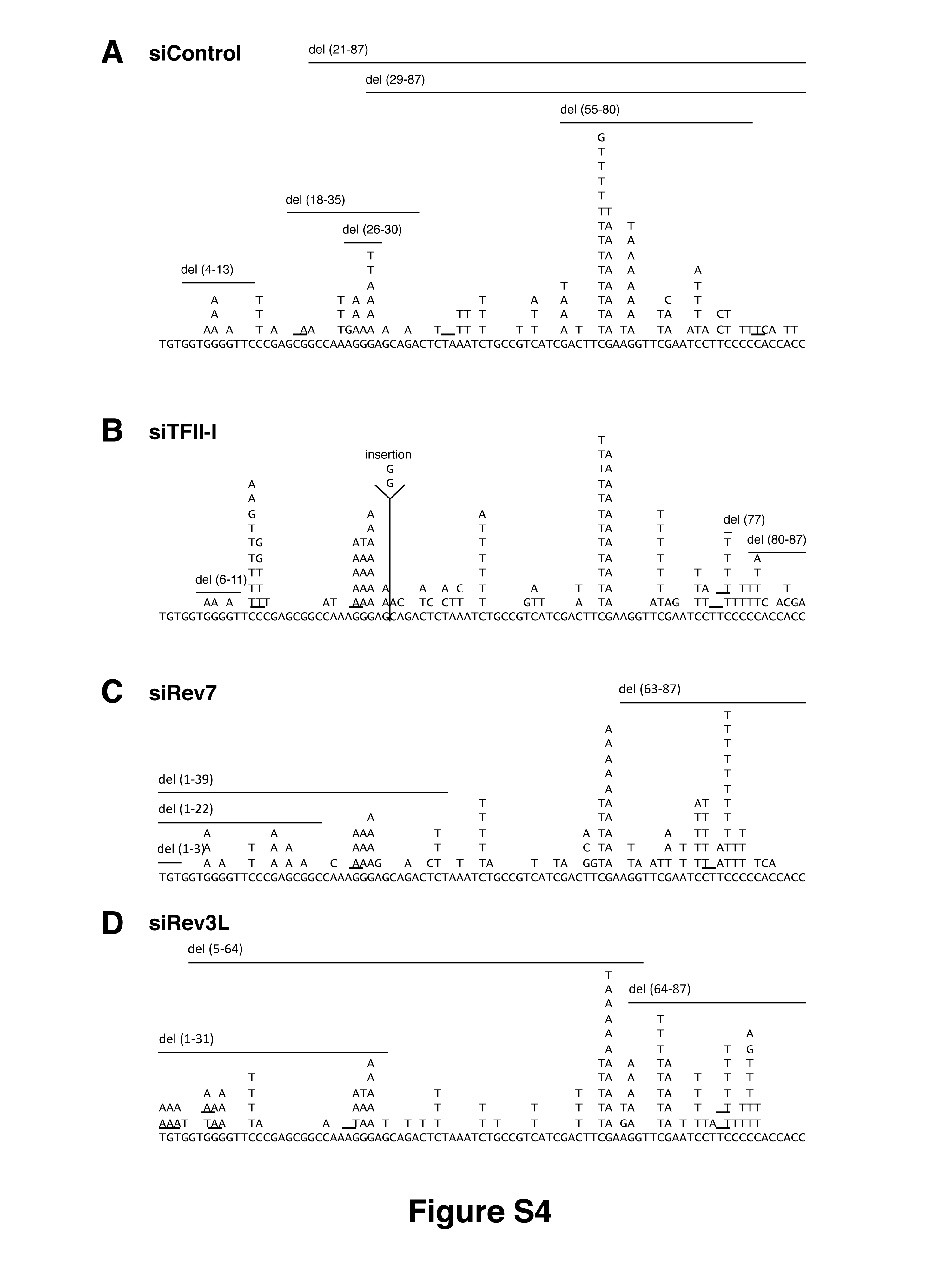

Supplement: Figure S4 — Depletion of TFII-I or Pol ζ does not alter the TLS mutation spectrum. (A-D) Position and type of mutations in the UV-irradiated SupF gene recovered from 293T cells transfected with the indicated siRNAs. (JPG) [file pgen.1004419.s004.jpg]

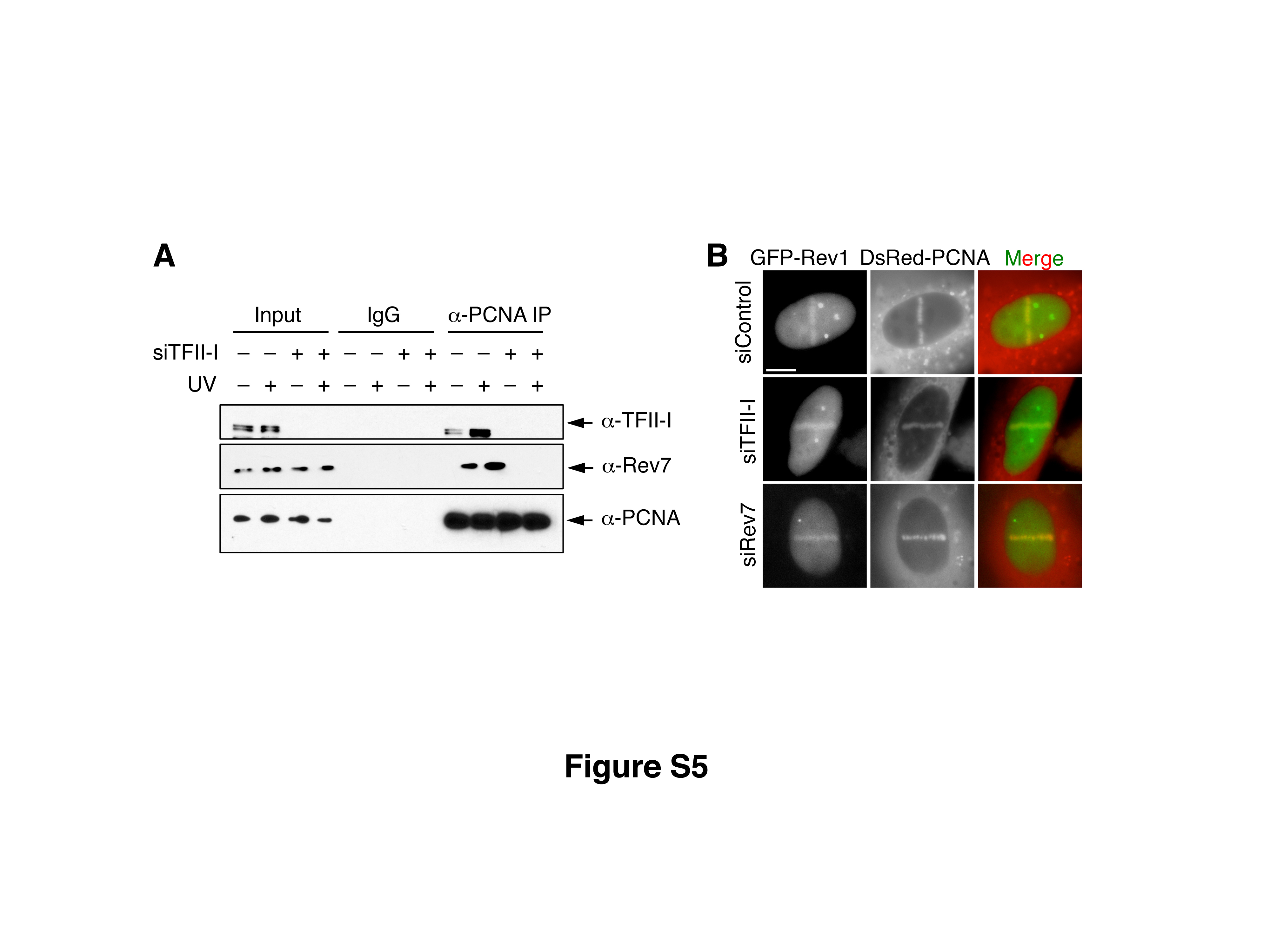

Supplement: Figure S5 — TFII-I is required for the PCNA–Rev7 interaction, but is dispensable for Rev1 recruitment to laser-induced DNA damage sites. (A) U2OS cells were mock transfected or transfected with siTFII-I, left untreated or irradiated with UV (60 J/m2), and treated with formaldehyde. Lysates and anti-PCNA IP of these cells were blotted with the indicated antibodies. (B) U2OS cells were transfected with GFP-Rev1 and DsRed-PCNA and the indicated siRNAs, and micro-irradiated with a 365-nm laser along straight lines. The GFP and DsRed channels are shown separately in gray scale and together in the merge. Scale bar, 10 µm. (JPG) [file pgen.1004419.s005.jpg]
