# Supplementary material for: The Transcription Factor TFII-I Promotes DNA Translesion Synthesis and Genomic Stability
Source: PLoS Genet. 2014 Jun 12;10(6):e1004419. doi: 10.1371/journal.pgen.1004419 (PMC4055408; doi:10.1371/journal.pgen.1004419)
Supplement: Table S1 — Mass spectrometry analysis of TAP-Rev7 binding proteins. (PDF) [file pgen.1004419.s006.pdf]

Table S1 Mass spectrometry analysis of TAP-Rev7 binding proteins

								SPECTRAL COUNTS							
**Protein**	**Description**	**Indistinguishable Protein**	**Prob**	**PSMs**	**Peptides**	**% Coverage**	**Modifications**	**1**	**2**	**3**	**4**	**5**	**6**	**7**	**8**
tr|B4DH52|B4DH52_HUMAN	Uncharacterized protein OS=Homo sapiens GN=GTF2I PE=2 SV=1		1	534	49	52.4	Gln->pyro-Glu (N-term Q),Oxidation (M)	3.96	38.61	199	113.86	161.38	26.72	21.78	14.84
sp|P27708|PYR1_HUMAN	CAD protein OS=Homo sapiens GN=CAD PE=1 SV=3	tr|F8VPD4|F8VPD4_HUMA	1	30	22	13.6	Oxidation (M)	29	2						1
sp|P04264|K2C1_HUMAN	Keratin, type II cytoskeletal 1 OS=Homo sapiens GN=KRT1 PE=1 SV=6		1	117	22	37.7	Gln->pyro-Glu (N-term Q),Oxidation (M)	17.5	11.5	10.5	18.5	22.5	19.5	9.5	13.5
sp|P35908|K22E_HUMAN	Keratin, type II cytoskeletal 2 epidermal OS=Homo sapiens GN=KRT2 PE=1 SV=2		1	59	19	42.9		6	6	3.99	10	16	11	2	4
sp|Q96JM3|ZN828_HUMAN	Zinc finger protein 828 OS=Homo sapiens GN=ZNF828 PE=1 SV=2		1	43	18	26.2	Oxidation (M)			12	26	5	1		
sp|P11021|GRP78_HUMAN	78 kDa glucose-regulated protein OS=Homo sapiens GN=HSPA5 PE=1 SV=2		1	14	15	32	Oxidation (M)					14			
sp|P13645|K1C10_HUMAN	Keratin, type I cytoskeletal 10 OS=Homo sapiens GN=KRT10 PE=1 SV=6		1	94	15	31	Oxidation (M)	14	7	10	13	14	17	7	13
sp|Q7Z3K3-2|POGZ_HUMAN	Isoform 2 of Pogo transposable element with ZNF domain OS=Homo sapiens GN=	sp|Q7Z3K3-3|POGZ_HUM/	1	31	13	13.8	Oxidation (M)		6.99		6.98	6	11		
sp|P35527|K1C9_HUMAN	Keratin, type I cytoskeletal 9 OS=Homo sapiens GN=KRT9 PE=1 SV=3		1	91	12	32.7	Oxidation (M)	13	10	7	14	16	20	6	10
sp|P68371|TBB4B_HUMAN	Tubulin beta-4B chain OS=Homo sapiens GN=TUBB4B PE=1 SV=1		1	27	12	32.8	Oxidation (M)				0.96		10.47	10.44	3.81
sp|O00410-3|IPO5_HUMAN	Isoform 3 of Importin-5 OS=Homo sapiens GN=IPO5	sp|O00410|IPO5_HUMAN;	1	29	11	13.8	Oxidation (M)	0.99	0.99	1.98	17.79	5.94	1.98	0.99	0.98
sp|O60884|DNJA2_HUMAN	DnaJ homolog subfamily A member 2 OS=Homo sapiens GN=DNAJA2 PE=1 SV=1		1	28	10	26.2	Oxidation (M)							28	
sp|P31689|DNJA1_HUMAN	DnaJ homolog subfamily A member 1 OS=Homo sapiens GN=DNAJA1 PE=1 SV=2		1	21	10	28.2	Gln->pyro-Glu (N-term Q),Oxidation (M)							23	
sp|P42704|LPPRC_HUMAN	Leucine-rich PPR motif-containing protein, mitochondrial OS=Homo sapiens GN=LRPPRC PE=1 SV=3		1	13	10	9.7	Oxidation (M)			12.98					
sp|Q9NVI1|FANCI_HUMAN	Fanconi anemia group I protein OS=Homo sapiens GN=FANCI PE=1 SV=4		1	11	9	10.5	Oxidation (M)		10.97						
sp|P10809|CH60_HUMAN	60 kDa heat shock protein, mitochondrial OS=Homo sapiens GN=HSPD1 PE=1 SV=2		1	11	9	26	Oxidation (M)						10.93		
sp|P55072|TERA_HUMAN	Transitional endoplasmic reticulum ATPase OS=Homo sapiens GN=VCP PE=1 SV=4		1	18	9	16.3	Oxidation (M)				11	2	6		
sp|P16615-2|AT2A2_HUMAN	Isoform 2 of Sarcoplasmic/endoplasmic reticulum calcium ATPase 2 OS=Homo sap	sp|P16615-3|AT2A2_HUM/	1	9	9	12.6	Oxidation (M)				8.9				
sp|Q9BQE3|TBA1C_HUMAN	Tubulin alpha-1C chain OS=Homo sapiens GN=TUBA1C PE=1 SV=1	tr|F5H5D3|F5H5D3_HUMA	1	72	9	34.1			0.98		3.86	3.86	35.62	14.48	12.52
sp|O00231|PSD11_HUMAN	26S proteasome non-ATPase regulatory subunit 11 OS=Homo sapiens GN=PSMD11 PE=1 SV=3		1	13	8	24.4	Oxidation (M)							10	3
sp|P54652|HSP72_HUMAN	Heat shock-related 70 kDa protein 2 OS=Homo sapiens GN=HSPA2 PE=1 SV=1		1	6	8	14.4	Oxidation (M)					5.85			0.98
sp|Q92616|GCN1L_HUMAN	Translational activator GCN1 OS=Homo sapiens GN=GCN1L1 PE=1 SV=6		1	8	8	4.9	Oxidation (M)	8							
sp|O14980|XPO1_HUMAN	Exportin-1 OS=Homo sapiens GN=XPO1 PE=1 SV=1		1	14	8	10.8	Oxidation (M)				14				
sp|Q00839-2|HNRPU_HUMAN	Isoform Short of Heterogeneous nuclear ribonucleoprotein U OS=Homo sapiens G	sp|Q00839|HNRPU_HUMA	1	16	8	11.5	Oxidation (M)			5	10	1	1	1	
sp|P08238|HS90B_HUMAN	Heat shock protein HSP 90-beta OS=Homo sapiens GN=HSP90AB1 PE=1 SV=4		1	8	7	12.7	Oxidation (M)					7		1	
sp|P22626-2|ROA2_HUMAN	Isoform A2 of Heterogeneous nuclear ribonucleoproteins A2/B1 OS=Homo sapiens	sp|P22626|ROA2_HUMAN;	1	9	7	36.4	Oxidation (M)								9
sp|P02533|K1C14_HUMAN	Keratin, type I cytoskeletal 14 OS=Homo sapiens GN=KRT14 PE=1 SV=4		1	13	7	18.9	Oxidation (M)	3		1	2	1		4	2
tr|B4DM67|B4DM67_HUMAN	Uncharacterized protein OS=Homo sapiens GN=CSE1L PE=2 SV=1		1	6	7	14.5	Oxidation (M)				5.95				
sp|P05023|AT1A1_HUMAN	Sodium/potassium-transporting ATPase subunit alpha-1 OS=Homo sapiens GN=A1	tr|B7Z3U6|B7Z3U6_HUMAI	1	6	7	10.8	Gln->pyro-Glu (N-term Q),Oxidation (M)				5.9				
sp|P60709|ACTB_HUMAN	Actin, cytoplasmic 1 OS=Homo sapiens GN=ACTB PE=1 SV=1	sp|P63261|ACTG_HUMAN	1	33	6	27.3	Gln->pyro-Glu (N-term Q),Oxidation (M)	1.95		0.96	2.91	3.9	3.87	1.98	17.4
sp|P78371|TCPB_HUMAN	T-complex protein 1 subunit beta OS=Homo sapiens GN=CCT2 PE=1 SV=4	tr|B7ZAT2|B7ZAT2_HUMAI	1	6	6	21.1	Oxidation (M)						6		
sp|Q9Y4W6|AFG32_HUMAN	AFG3-like protein 2 OS=Homo sapiens GN=AFG3L2 PE=1 SV=2		1	5	5	8	Oxidation (M)					5			
sp|P08779|K1C16_HUMAN	Keratin, type I cytoskeletal 16 OS=Homo sapiens GN=KRT16 PE=1 SV=4		1	2	5	13.7	Oxidation (M)							2	
sp|O75155-2|CAND2_HUMAN	Isoform 2 of Cullin-associated NEDD8-dissociated protein 2 OS=Homo sapiens GN	sp|O75155|CAND2_HUMA	1	5	5	6	Oxidation (M)			5					
sp|O75306|NDUS2_HUMAN	NADH dehydrogenase [ubiquinone] iron-sulfur protein 2, mitochondrial OS=Homo sapiens GN=NDUFS2 PE=1		1	17	5	17.5	Oxidation (M)							9	9
sp|O95831-3|AIFM1_HUMAN	Isoform 3 of Apoptosis-inducing factor 1, mitochondrial OS=Homo sapiens GN=AIF	sp|O95831|AIFM1_HUMAN	1	6	5	10.8	Oxidation (M)						5.91		
sp|Q14257|RCN2_HUMAN	Reticulocalbin-2 OS=Homo sapiens GN=RCN2 PE=1 SV=1	tr|F8WCY5|F8WCY5_HUM	1	20	5	19.9	Gln->pyro-Glu (N-term Q)							19	2
sp|Q3ZCQ8-2|TIM50_HUMAN	Isoform 2 of Mitochondrial import inner membrane translocase subunit TIM50 OS=I	sp|Q3ZCQ8|TIM50_HUMAI	1	11	5	17.6									11
sp|P38646|GRP75_HUMAN	Stress-70 protein, mitochondrial OS=Homo sapiens GN=HSPA9 PE=1 SV=2	tr|B7Z4V2|B7Z4V2_HUMAI	1	6	5	11.4						5.94			
sp|P50990|TCPQ_HUMAN	T-complex protein 1 subunit theta OS=Homo sapiens GN=CCT8 PE=1 SV=4	tr|B4DEM7|B4DEM7_HUM/	1	5	5	10.4	Oxidation (M)						5		
sp|P07900-2|HS90A_HUMAN	Isoform 2 of Heat shock protein HSP 90-alpha OS=Homo sapiens GN=HSP90AA1	sp|P07900|HS90A_HUMAN	1	3	4	11.9						2		1	
sp|O43242|PSMD3_HUMAN	26S proteasome non-ATPase regulatory subunit 3 OS=Homo sapiens GN=PSMD3	tr|B4DT72|B4DT72_HUMAI	1	5	4	14.6	Oxidation (M)						5		
sp|Q9UJ14|GGT7_HUMAN	Gamma-glutamyltransferase 7 OS=Homo sapiens GN=GGT7 PE=1 SV=2		1	6	4	7.9	Oxidation (M)				0.99	4.98			
sp|Q99832|TCPH_HUMAN	T-complex protein 1 subunit eta OS=Homo sapiens GN=CCT7 PE=1 SV=2	tr|A8MWI8|A8MWI8_HUMA	1	5	4	16.2	Oxidation (M)						5		
sp|P51991-2|ROA3_HUMAN	Isoform 2 of Heterogeneous nuclear ribonucleoprotein A3 OS=Homo sapiens GN=I	sp|P51991|ROA3_HUMAN;	1	4	4	15.8	Oxidation (M)								4
sp|P53396|ACLY_HUMAN	ATP-citrate synthase OS=Homo sapiens GN=ACLY PE=1 SV=3	tr|B4E3P0|B4E3P0_HUMAI	1	5	4	7.2	Oxidation (M)				4		1		
sp|Q13263-2|TIF1B_HUMAN	Isoform 2 of Transcription intermediary factor 1-beta OS=Homo sapiens GN=TRIM	sp|Q13263|TIF1B_HUMAN	1	9	4	8	Oxidation (M)				6	3			
sp|P21281|VATB2_HUMAN	V-type proton ATPase subunit B, brain isoform OS=Homo sapiens GN=ATP6V1B2 PE=1 SV=3		1	3	4	10.4	Oxidation (M)						3		
sp|Q13724|MOGS_HUMAN	Mannosyl-oligosaccharide glucosidase OS=Homo sapiens GN=MOGS PE=1 SV=5	tr|F5H6D0|F5H6D0_HUMA	1	3	4	7.8						2.99			
sp|P28482|MK01_HUMAN	Mitogen-activated protein kinase 1 OS=Homo sapiens GN=MAPK1 PE=1 SV=3	tr|A8CZ64|A8CZ64_HUMAI	1	5	4	18.4	Oxidation (M)								4.99
sp|P35998|PRS7_HUMAN	26S protease regulatory subunit 7 OS=Homo sapiens GN=PSMC2 PE=1 SV=3		1	5	4	12.9	Oxidation (M)							5	
sp|P40939|ECHA_HUMAN	Trifunctional enzyme subunit alpha, mitochondrial OS=Homo sapiens GN=HADHA PE=1 SV=2		1	4	4	8.5	Oxidation (M)					4			
sp|Q9NTJ4|MA2C1_HUMAN	Alpha-mannosidase 2C1 OS=Homo sapiens GN=MAN2C1 PE=1 SV=1		1	3	3	3.6	Oxidation (M)				3				
sp|P56192|SYMC_HUMAN	Methionyl-tRNA synthetase, cytoplasmic OS=Homo sapiens GN=MARS PE=1 SV=	tr|A6NC17|A6NC17_HUMA	1	3	3	6.5	Oxidation (M)				3				
sp|P09651-2|ROA1_HUMAN	Isoform A1-A of Heterogeneous nuclear ribonucleoprotein A1 OS=Homo sapiens G	sp|P09651-3|ROA1_HUMA	1	4	3	16.5									3.96
sp|Q96QK1|VPS35_HUMAN	Vacuolar protein sorting-associated protein 35 OS=Homo sapiens GN=VPS35 PE=	tr|F5GYF5|F5GYF5_HUMA	1	6	3	6						4	2		
sp|Q99536|VAT1_HUMAN	Synaptic vesicle membrane protein VAT-1 homolog OS=Homo sapiens GN=VAT1 F	tr|B0AZP7|B0AZP7_HUMA	1	3	3	17.4	Oxidation (M)							3	
sp|O95373|IPO7_HUMAN	Importin-7 OS=Homo sapiens GN=IPO7 PE=1 SV=1		1	2	3	4.6	Oxidation (M)				2				
sp|Q9UNM6|PSD13_HUMAN	26S proteasome non-ATPase regulatory subunit 13 OS=Homo sapiens GN=PSMD13 PE=1 SV=2		1	3	3	9.8	Oxidation (M)								2.97
sp|Q9UBX5|FBLN5_HUMAN	Fibulin-5 OS=Homo sapiens GN=FBLN5 PE=1 SV=1	tr|G3V4U0|G3V4U0_HUMA	1	5	3	9.2	Gln->pyro-Glu (N-term Q),Oxidation (M)						4	1	
sp|Q9Y3I0|RTCB_HUMAN	tRNA-splicing ligase RtcB homolog OS=Homo sapiens GN=C22orf28 PE=1 SV=1	tr|E9PCW3|E9PCW3_HUM	1	3	3	7.9	Oxidation (M)						3		
sp|Q9UJZ1|STML2_HUMAN	Stomatin-like protein 2 OS=Homo sapiens GN=STOML2 PE=1 SV=1	tr|B4E1K7|B4E1K7_HUMAI	1	3	3	14.8									3
sp|Q9Y4R8|TELO2_HUMAN	Telomere length regulation protein TEL2 homolog OS=Homo sapiens GN=TELO2 PE=1 SV=2		1	2	3	4.5	Oxidation (M)				2	1			
sp|Q9Y6G9|DC1L1_HUMAN	Cytoplasmic dynein 1 light intermediate chain 1 OS=Homo sapiens GN=DYNC1LI1	tr|E9PHI6|E9PHI6_HUMAN	1	3	3	12.3	Oxidation (M)						3		
sp|P35222|CTNB1_HUMAN	Catenin beta-1 OS=Homo sapiens GN=CTNNB1 PE=1 SV=1	tr|B4DGU4|B4DGU4_HUM/	1	6	3	5.5	Oxidation (M)				6				
sp|Q00610-2|CLH1_HUMAN	Isoform 2 of Clathrin heavy chain 1 OS=Homo sapiens GN=CLTC	sp|Q00610|CLH1_HUMAN;	1	2	3	3.2	Oxidation (M)		2						
sp|O00571|DDX3X_HUMAN	ATP-dependent RNA helicase DDX3X OS=Homo sapiens GN=DDX3X PE=1 SV=3	tr|B4E3E8|B4E3E8_HUMAI	1	5	3	6.5						3.98	1		
sp|O75131|CPNE3_HUMAN	Copine-3 OS=Homo sapiens GN=CPNE3 PE=1 SV=1		1	2	3	7.3							2		
sp|P02786|TFR1_HUMAN	Transferrin receptor protein 1 OS=Homo sapiens GN=TFRC PE=1 SV=2	tr|G3V0E5|G3V0E5_HUMA	1	3	3	6.3						3			
sp|P13647|K2C5_HUMAN	Keratin, type II cytoskeletal 5 OS=Homo sapiens GN=KRT5 PE=1 SV=3	tr|E7EU87|E7EU87_HUMA	0.9997	5	3	6.1	Oxidation (M)	1				2	2		1
sp|P17980|PRS6A_HUMAN	26S protease regulatory subunit 6A OS=Homo sapiens GN=PSMC3 PE=1 SV=3	tr|E9PKD5|E9PKD5_HUMA	1	4	3	18.1	Oxidation (M)							3.96	
sp|P17987|TCPA_HUMAN	T-complex protein 1 subunit alpha OS=Homo sapiens GN=TCP1 PE=1 SV=1		1	4	3	10.4	Oxidation (M)						3.96		
sp|Q14974|IMB1_HUMAN	Importin subunit beta-1 OS=Homo sapiens GN=KPNB1 PE=1 SV=2		1	5	3	4.6	Oxidation (M)				2.98	2.97			
sp|Q15008|PSMD6_HUMAN	26S proteasome non-ATPase regulatory subunit 6 OS=Homo sapiens GN=PSMD6	tr|C9IZE4|C9IZE4_HUMAN	1	5	3	11.4	Oxidation (M)							1	4
sp|P06733|ENOA_HUMAN	Alpha-enolase OS=Homo sapiens GN=ENO1 PE=1 SV=2		1	4	3	12.4	Oxidation (M)							4	
sp|Q86V20-2|FA35A_HUMAN	Isoform 2 of Protein FAM35A OS=Homo sapiens GN=FAM35A	sp|Q86V20|FA35A_HUMAN	1	3	3	4.4	Oxidation (M)				3				
sp|Q8WXE1-2|ATRIP_HUMAN	Isoform 2 of ATR-interacting protein OS=Homo sapiens GN=ATRIP	sp|Q8WXE1-3|ATRIP_HUM	1	3	3	5.9						3			
sp|Q99460-2|PSMD1_HUMAN	Isoform 2 of 26S proteasome non-ATPase regulatory subunit 1 OS=Homo sapiens	sp|Q99460|PSMD1_HUMA	1	4	3	5.9	Oxidation (M)				4				
sp|Q99729-2|ROAA_HUMAN	Isoform 2 of Heterogeneous nuclear ribonucleoprotein A/B OS=Homo sapiens GN=	sp|Q99729-3|ROAA_HUMA	1	3	3	16.4	Oxidation (M)								3
sp|P46379-2|BAG6_HUMAN	Isoform 2 of Large proline-rich protein BAG6 OS=Homo sapiens GN=BAG6	sp|P46379-3|BAG6_HUMA	1	3	3	4.1	Oxidation (M)						3		
sp|P49368|TCPG_HUMAN	T-complex protein 1 subunit gamma OS=Homo sapiens GN=CCT3 PE=1 SV=4	tr|B4DUR8|B4DUR8_HUM/	1	3	3	8.4	Oxidation (M)						2.97		

sp|P53618|COPB_HUMAN	Coatomer subunit beta OS=Homo sapiens GN=COPB1 PE=1 SV=3		1	3	3	4.8	Oxidation (M)				3				
sp|P55209|NP1L1_HUMAN	Nucleosome assembly protein 1-like 1 OS=Homo sapiens GN=NAP1L1 PE=1 SV=	tr|B3KNT8|B3KNT8_HUMA	1	4	3	35.1	Oxidation (M)						3.95		
sp|Q9H4M9|EHD1_HUMAN	EH domain-containing protein 1 OS=Homo sapiens GN=EHD1 PE=1 SV=2	tr|C9J2Z4|C9J2Z4_HUMAN	1	3	2	8.5	Oxidation (M)						2	1	
sp|O14654|IRS4_HUMAN	Insulin receptor substrate 4 OS=Homo sapiens GN=IRS4 PE=1 SV=1		1	4	2	2.2	Oxidation (M)				2	2			
sp|O14929-2|HAT1_HUMAN	Isoform B of Histone acetyltransferase type B catalytic subunit OS=Homo sapiens (	sp|O14929|HAT1_HUMAN;	0.9984	1	2	8.7	Oxidation (M)							1	
sp|P40938|RFC3_HUMAN	Replication factor C subunit 3 OS=Homo sapiens GN=RFC3 PE=1 SV=2	tr|A8MX32|A8MX32_HUMA	0.9512	1	2	9.3									1
sp|Q96P70|IPO9_HUMAN	Importin-9 OS=Homo sapiens GN=IPO9 PE=1 SV=3		1	2	2	3.5	Oxidation (M)				2				
sp|O94788-2|AL1A2_HUMAN	Isoform 2 of Retinal dehydrogenase 2 OS=Homo sapiens GN=ALDH1A2	sp|O94788|AL1A2_HUMAN	1	2	2	8.5	Oxidation (M)						2		
sp|Q14697-2|GANAB_HUMAN	Isoform 2 of Neutral alpha-glucosidase AB OS=Homo sapiens GN=GANAB	sp|Q14697|GANAB_HUMA	0.738	1	2	3.1	Oxidation (M)				1				
sp|P04632|CPNS1_HUMAN	Calpain small subunit 1 OS=Homo sapiens GN=CAPNS1 PE=1 SV=1		1	8	2	10.4	Oxidation (M)				2	2	2	2	2
sp|P62333|PRS10_HUMAN	26S protease regulatory subunit 10B OS=Homo sapiens GN=PSMC6 PE=1 SV=1		0.6888	1	2	6.4	Oxidation (M)								1
sp|Q9H0D6-2|XRN2_HUMAN	Isoform 2 of 5'-3' exoribonuclease 2 OS=Homo sapiens GN=XRN2	sp|Q9H0D6|XRN2_HUMAN	1	2	2	3.1	Oxidation (M)				2				
sp|P0CG47|UBB_HUMAN	Polyubiquitin-B OS=Homo sapiens GN=UBB PE=1 SV=1	sp|P0CG48|UBC_HUMAN;	1	7	2	38					2	1	2	1	1
sp|P15735-2|PHKG2_HUMAN	Isoform 2 of Phosphorylase b kinase gamma catalytic chain, testis/liver isoform OS	sp|P15735|PHKG2_HUMAI	1	2	2	8	Oxidation (M)								2
sp|Q9Y230|RUVB2_HUMAN	RuvB-like 2 OS=Homo sapiens GN=RUVBL2 PE=1 SV=3	tr|B3KQ59|B3KQ59_HUMA	1	2	2	6.9	Oxidation (M)							2	
sp|P17655|CAN2_HUMAN	Calpain-2 catalytic subunit OS=Homo sapiens GN=CAPN2 PE=1 SV=6	tr|B7ZA96|B7ZA96_HUMAI	1	2	2	4.5	Oxidation (M)					2			
sp|O43592|XPOT_HUMAN	Exportin-T OS=Homo sapiens GN=XPOT PE=1 SV=2		0.9984	1	2	2.7	Oxidation (M)				1				
sp|P52597|HNRPF_HUMAN	Heterogeneous nuclear ribonucleoprotein F OS=Homo sapiens GN=HNRNPF PE=1 SV=3		0.9984	1	2	8								1	
sp|P22695|QCR2_HUMAN	Cytochrome b-c1 complex subunit 2, mitochondrial OS=Homo sapiens GN=UQCRC2 PE=1 SV=3		1	2	2	7.1									2
sp|Q13409-2|DC1I2_HUMAN	Isoform 2B of Cytoplasmic dynein 1 intermediate chain 2 OS=Homo sapiens GN=D	sp|Q13409-3|DC1I2_HUMA	0.9829	2	2	5.2	Oxidation (M)					1.99			
sp|P60842|IF4A1_HUMAN	Eukaryotic initiation factor 4A-I OS=Homo sapiens GN=EIF4A1 PE=1 SV=1	sp|Q14240-2|IF4A2_HUMA	0.9998	2	2	8	Oxidation (M)							1.98	
sp|P62191|PRS4_HUMAN	26S protease regulatory subunit 4 OS=Homo sapiens GN=PSMC1 PE=1 SV=1	tr|B4DR63|B4DR63_HUMA	1	2	2	9.8	Oxidation (M)						2		
sp|P68104|EF1A1_HUMAN	Elongation factor 1-alpha 1 OS=Homo sapiens GN=EEF1A1 PE=1 SV=1	sp|Q5VTE0|EF1A3_HUMAI	0.9999	2	2	5.9								1.98	
sp|P35606|COPB2_HUMAN	Coatomer subunit beta' OS=Homo sapiens GN=COPB2 PE=1 SV=2	tr|B4DZI8|B4DZI8_HUMAN	1	4	2	3.3					4				
sp|P50991|TCPD_HUMAN	T-complex protein 1 subunit delta OS=Homo sapiens GN=CCT4 PE=1 SV=4	tr|B7Z2F4|B7Z2F4_HUMAN	1	2	2	8.5	Oxidation (M)						2		
sp|Q6P3X3|TTC27_HUMAN	Tetratricopeptide repeat protein 27 OS=Homo sapiens GN=TTC27 PE=1 SV=1		1	2	2	3.3	Oxidation (M)					2			
sp|Q8TEX9-2|IPO4_HUMAN	Isoform 2 of Importin-4 OS=Homo sapiens GN=IPO4	sp|Q8TEX9|IPO4_HUMAN;	1	5	2	3.2	Oxidation (M)			4	1				
sp|P11177-2|ODPB_HUMAN	Isoform 2 of Pyruvate dehydrogenase E1 component subunit beta, mitochondrial O	sp|P11177|ODPB_HUMAN;	0.9999	2	2	9.4	Oxidation (M)								1.98
sp|O15397|IPO8_HUMAN	Importin-8 OS=Homo sapiens GN=IPO8 PE=1 SV=2		1	2	2	2.3					2				
sp|Q9UBL6-2|CPNE7_HUMAN	Isoform 2 of Copine-7 OS=Homo sapiens GN=CPNE7	sp|Q9UBL6|CPNE7_HUMA	0.9989	1	2	4.3							1		
sp|O75436|VP26A_HUMAN	Vacuolar protein sorting-associated protein 26A OS=Homo sapiens GN=VPS26A P	tr|F5H4L7|F5H4L7_HUMAN	1	2	2	14.4	Oxidation (M)								2
sp|P30153|2AAA_HUMAN	Serine/threonine-protein phosphatase 2A 65 kDa regulatory subunit A alpha isoforn	tr|B4DDF7|B4DDF7_HUMA	0.9999	3	2	5.7	Oxidation (M)						3		
sp|Q5SQN1|SNP47_HUMAN	Synaptosomal-associated protein 47 OS=Homo sapiens GN=SNAP47 PE=2 SV=3		0.9999	2	2	6.2								2	
sp|O00311|CDC7_HUMAN	Cell division cycle 7-related protein kinase OS=Homo sapiens GN=CDC7 PE=1 SV=1		0.9998	2	2	4.4	Oxidation (M)						2		
sp|Q6XZF7-2|DNMBP_HUMAN	Isoform 2 of Dynamin-binding protein OS=Homo sapiens GN=DNMBP	sp|Q6XZF7|DNMBP_HUMA	0.9998	2	2	5.8			2						
sp|P55084|ECHB_HUMAN	Trifunctional enzyme subunit beta, mitochondrial OS=Homo sapiens GN=HADHB F	tr|B4E2W0|B4E2W0_HUMA	0.9972	2	2	7.1	Oxidation (M)							1.98	
sp|Q13547|HDAC1_HUMAN	Histone deacetylase 1 OS=Homo sapiens GN=HDAC1 PE=1 SV=1	sp|Q92769|HDAC2_HUMA	0.9996	2	2	41.7	Oxidation (M)						2		
sp|Q96JJ7|TMX3_HUMAN	Protein disulfide-isomerase TMX3 OS=Homo sapiens GN=TMX3 PE=1 SV=2		0.9966	2	2	6.4	Oxidation (M)						1.99		
sp|Q8IY67-2|RAVR1_HUMAN	Isoform 2 of Ribonucleoprotein PTB-binding 1 OS=Homo sapiens GN=RAVER1	sp|Q8IY67|RAVR1_HUMAN	0.9967	2	2	5.8						1.99			
sp|Q9BXW9-2|FACD2_HUMAN	Isoform 2 of Fanconi anemia group D2 protein OS=Homo sapiens GN=FANCD2	sp|Q9BXW9-3|FACD2_HUI	0.9971	2	2	2.4			1.99						
sp|Q9UNF1-2|MAGD2_HUMAN	Isoform 2 of Melanoma-associated antigen D2 OS=Homo sapiens GN=MAGED2	sp|Q9UNF1|MAGD2_HUMA	0.9994	2	2	4.8						1.5			
sp|Q9Y5V3-2|MAGD1_HUMAN	Isoform 2 of Melanoma-associated antigen D1 OS=Homo sapiens GN=MAGED1	sp|Q9Y5V3|MAGD1_HUMA	0.9994	2	2	3.5					1	0.5			
sp|Q9BRK5-3|CAB45_HUMAN	Isoform 3 of 45 kDa calcium-binding protein OS=Homo sapiens GN=SDF4	sp|Q9BRK5-4|CAB45_HUN	0.9994	2	2	12.4	Oxidation (M)							2	
sp|P07814|SYEP_HUMAN	Bifunctional aminoacyl-tRNA synthetase OS=Homo sapiens GN=EPRS PE=1 SV=5		0.9985	1	2	2.2			1						
sp|P22314|UBA1_HUMAN	Ubiquitin-like modifier-activating enzyme 1 OS=Homo sapiens GN=UBA1 PE=1 SV=3		0.9985	1	2	3.1					1				
sp|Q15233|NONO_HUMAN	Non-POU domain-containing octamer-binding protein OS=Homo sapiens GN=NON	tr|F5GYZ3|F5GYZ3_HUMA	1	2	2	7.6	Oxidation (M)						2		
sp|P26641|EF1G_HUMAN	Elongation factor 1-gamma OS=Homo sapiens GN=EEF1G PE=1 SV=3	tr|B4DTG2|B4DTG2_HUMA	1	2	2	6.2								2	
sp|Q16531|DDB1_HUMAN	DNA damage-binding protein 1 OS=Homo sapiens GN=DDB1 PE=1 SV=1	tr|B4DZP5|B4DZP5_HUMA	0.9999	2	2	4.1	Oxidation (M)				1.98				
sp|P08195-2|4F2_HUMAN	Isoform 2 of 4F2 cell-surface antigen heavy chain OS=Homo sapiens GN=SLC3A2	sp|P08195-3|4F2_HUMAN;	0.9999	2	2	5.3						1.98			
sp|Q8N163-2|K1967_HUMAN	Isoform 2 of Protein KIAA1967 OS=Homo sapiens GN=KIAA1967	sp|Q8N163|K1967_HUMAN	1	2	2	12.6				2					
sp|Q96EY1|DNJA3_HUMAN	DnaJ homolog subfamily A member 3, mitochondrial OS=Homo sapiens GN=DNAJA3 PE=1 SV=2		1	3	2	6	Oxidation (M)								3
sp|P43243|MATR3_HUMAN	Matrin-3 OS=Homo sapiens GN=MATR3 PE=1 SV=2	tr|A8MXP9|A8MXP9_HUMA	0.9999	2	2	4.3					1.98				
sp|P48444|COPD_HUMAN	Coatomer subunit delta OS=Homo sapiens GN=ARCN1 PE=1 SV=1	tr|B0YIW6|B0YIW6_HUMA	0.9999	2	2	4.9	Oxidation (M)						1.98		
sp|Q9BY77-2|PDIP3_HUMAN	Isoform 2 of Polymerase delta-interacting protein 3 OS=Homo sapiens GN=POLDII	sp|Q9BY77|PDIP3_HUMAN	1	2	2	8.4								2	
